# Robust Dipolar Layers between Organic Semiconductors and Silver for Energy-Level Alignment

**DOI:** 10.1021/acsami.3c18697

**Published:** 2024-03-29

**Authors:** Tomáš Krajňák, Veronika Stará, Pavel Procházka, Jakub Planer, Tomáš Skála, Matthias Blatnik, Jan Čechal

**Affiliations:** † CEITEC—Central European Institute of Technology, Brno University of Technology, Purkyňova 123, 612 00 Brno, Czech Republic; ‡ Department of Surface and Plasma Science, Faculty of Mathematics and Physics, Charles University, V Holešovičkách 2, 180 00 Prague 8, Czech Republic; § Institute of Physical Engineering, Brno University of Technology, Technická 2896/2, 616 69 Brno, Czech Republic

**Keywords:** charge injection layers, self-assembly, surfaces, photoelectron spectroscopy, energy levels, low-energy electron microscopy, scanning tunneling microscopy

## Abstract

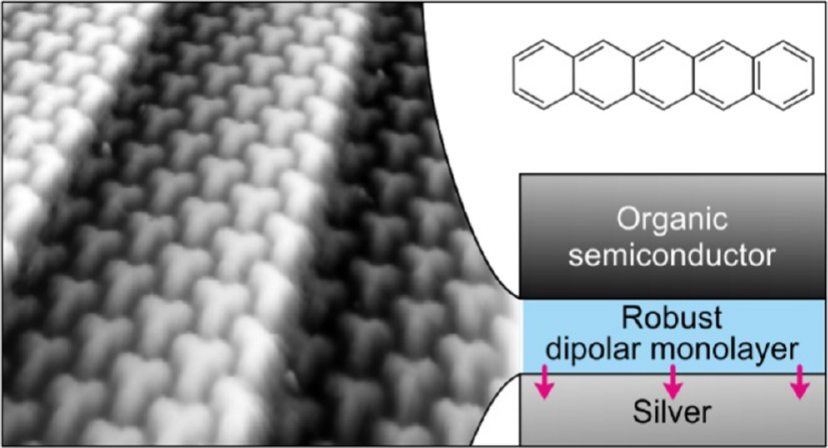

The interface between a metal electrode and an organic semiconductor (OS) layer has a defining role in the properties of the resulting device. To obtain the desired performance, interlayers are introduced to modify the adhesion and growth of OS and enhance the efficiency of charge transport through the interface. However, the employed interlayers face common challenges, including a lack of electric dipoles to tune the mutual position of energy levels, being too thick for efficient electronic transport, or being prone to intermixing with subsequently deposited OS layers. Here, we show that monolayers of 1,3,5-tris(4-carboxyphenyl)benzene (BTB) with fully deprotonated carboxyl groups on silver substrates form a compact layer resistant to intermixing while capable of mediating energy-level alignment and showing a large insensitivity to substrate termination. Employing a combination of surface-sensitive techniques, i.e., low-energy electron microscopy and diffraction, X-ray photoelectron spectroscopy, and scanning tunneling microscopy, we have comprehensively characterized the compact layer and proven its robustness against mixing with the subsequently deposited organic semiconductor layer. Density functional theory calculations show that the robustness arises from a strong interaction of carboxylate groups with the Ag surface, and thus, the BTB in the first layer is energetically favored. Synchrotron radiation photoelectron spectroscopy shows that this layer displays considerable electrical dipoles that can be utilized for work function engineering and electronic alignment of molecular frontier orbitals with respect to the substrate Fermi level. Our work thus provides a widely applicable molecular interlayer and general insights necessary for engineering of charge injection layers for efficient organic electronics.

## Introduction

Organic electronics is a significant technology for displays and illumination.^1−3^ In other fields that utilize organic semiconductors (OSs), e.g., in organic thin-film transistors^4^ and organic photovoltaics,^5^ the large-scale industrial applications are still limited. The performance of fast-switching and high-power organic electronic devices, like OFETs, is often highly influenced by the contact resistance^6−9^ originating from the energy-level misalignment between a metal electrode and an OS layer.^7,10−12^

Introducing ordered dipolar layers at the metal–OS interface can tune the electrode work function (WF) and the interfacial energy-level alignment (ELA) with the OS frontier orbitals (highest occupied molecular orbital (HOMO) or lowest unoccupied molecular orbital (LUMO)).^13,14^ These so-called charge injection layers (CILs) can thus significantly reduce the contact resistance and increase the efficiency of the charge-carrier injection into the OS layer. In this respect, molecular layers exhibiting electric dipoles can act as CILs between metal electrodes and OS layers;^15,16^ the dipoles can be either intrinsic to the deposited molecules, formed due to the molecule–substrate charge transfer, or by changing the molecular conformation (e.g., its bending).^13^ The self-assembled monolayers (SAMs) were intensively studied in this respect.^13,15−18^ The introduction of polar segments into the backbone can provide the desired electric dipoles necessary for WF engineering,^18^ but the molecular chains also present a decoupling layer that contributes to the contact resistance between the metal substrate and the OS layer deposited on the top.^17,19−21^ In this respect, the OS monolayers demonstrated promising changes of the WF with respect to ELA;^10,13,14,22^ however, they are prone to interdiffusion or formation of mixed phases with subsequently deposited molecular layers.^10,14,23−33^ A sharp, uniform, and stable interface during the lifetime of the device is required for technological applications of efficient CILs.

**Figure 1:**
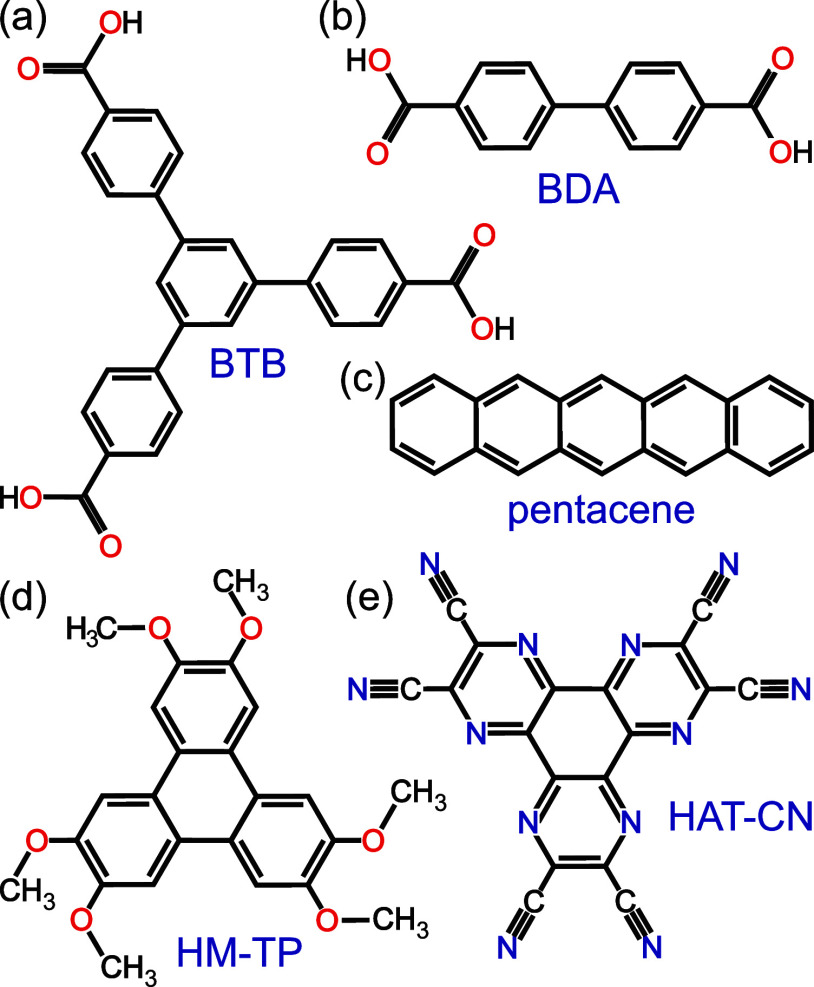
Chemical structure of organic molecules explored in this work. (a) 1,3,5-Tris(4-carboxyphenyl) benzene (BTB); (b) 4,4′-biphenyl dicarboxylic acid (BDA); (c) pentacene; (d) hexamethoxy-triphenylene (HM-TP); and (e) hexaazatriphenylene-hexacarbonitrile (HAT-CN).

Recently, we have shown that monolayers of aromatic carboxylic acids can act as CILs.^34^ In that system, the required electric dipoles are localized at the metal–organic interface, which results in removing the tunneling contact between the molecular layer and the metal electrode. However, the employed molecules share the main issue with other molecular species explored for this role: they readily mix with the deposited OS overlayer, which would compromise the performance of potential devices. Here, we show that 1,3,5-tris(4-carboxyphenyl) benzene (BTB, Figure 1a), an aromatic tricarboxylic acid, forms a robust layer that does not mix with deposited OS layers up to temperatures at which OSs either re-evaporate or BTB decompose.

The robust interface can be formed by employing molecules that strongly bind to the surface, like in SAMs. Concerning Ag surfaces, carboxyl-terminated SAMs^35−40^ show higher structural order than traditionally used thiol-based SAMs.^39,40^ Here, a partial charge transfer between molecule and substrate provides a physically robust and electronically strong connection,^9,12,15,17,41^ but intermixing with deposited porphyrin and phthalocyanine molecules even below room temperature was reported.^42^ In addition, the strong OS molecule–metal interaction induces undesirable changes to surface and OS film microstructure and substantial modification of interfacial electronic structure, which can profoundly impact contact and channel resistance and overall device performance.^7,12,19^ Some strongly interacting small organic molecules, like F4-TCNQ and F6-TCNNQ, may form an organometallic layer with silver with a thickness up to 50 nm, which is stable with respect to subsequent deposition of pentacene layers^43^ but is still far from an ideal case.

While providing favorable properties with respect to ELA, planar weakly adsorbing OS molecules are more prone to intermixing with subsequently deposited molecular layers. One of the possibilities is to change molecular functional groups or their number to strengthen organic–metal interaction and, thus, the first layer stability. In this respect, changing the molecular structure of pentacene oxo-derivatives from 6,13-pentacenequinone (P2O, featuring two oxygens) and 5,7,12,14-pentacenetetrone (P4O, 4 oxygens) leads to the change of adsorption behavior on Ag(111) from physisorption of P2O to chemisorption of P4O.^31^ In this case, the P4O layers were resistant to intermixing with subsequently deposited copper phthalocyanine (CuPc). The other possibility to obtain a semistable bilayer is to use 3,4,9,10-perylene-tetracarboxylic-dianhydride (PTCDA), which is stable against the mixing with subsequently deposited CuPc^44^ or tin phthalocyanine (SnPc).^25^ In these cases, a kinetic barrier exists regarding interlayer exchange in both CuPc/PTCDA/Ag and PTCDA/CuPc/Ag stacking orders, with a primary parameter governing stability at lower temperatures being the adsorption energy per area of the individual molecules.^30^ However, beyond the onset of desorption, the decisive parameter becomes the adsorption energy per molecule, and the preferred occupancy of the first layer can change.

Our previous study introduced aromatic carboxylic acids as dipolar layers.^34^ We have shown that the employed 4,4′-biphenyl dicarboxylic acid (BDA, Figure 1b) molecule can gradually deprotonate in direct contact with silver surfaces either thermally^45−48^ or by low-energy electrons,^49^ thus providing a possibility to finely tune the ELA. While considerable shifts in the WF and energy levels of deposited molecules up to 0.8 eV were induced, our later experiments have shown that it is prone to mix with pentacene layers deposited on top. In the present paper, we show that extending the molecule to three carboxylic groups results in a robust monolayer that does not mix with subsequently deposited OS molecules, i.e., pentacene (Figure 1c), a prototypical high mobility OS,^50^ HM-TP (Figure 1d), and HAT-CN (Figure 1e), an electron donor and acceptor, respectively. Our density functional theory (DFT) calculations show that the robustness is of a thermodynamic origin: the compact layer presents the lowest energy state. Thus, the molecular monolayers of fully deprotonated BTB form a viable platform on the path toward the ohmic contacts between electrodes on OS layers.

## Results and Discussion

We have performed experiments for two low-energy facets of the silver surface: Ag(111) and Ag(100). As the results are similar on both surfaces, we will focus our description on Ag(111) and give the results for the other facet in the Supporting Information. In the following, we will first show synchrotron radiation photoelectron spectroscopy results for gradual deprotonation of BTB and show that with respect to WF changes and ELA, the BTB behaves consistently with our earlier results on BDA.^34^ Then, we will discuss the obtained scanning tunneling microscopy (STM) and low-energy electron microscopy (LEEM) data for submonolayer and full monolayer coverages of the fully deprotonated molecule (marked as δ-BTB in the following), demonstrating that, contrary to BDA, the compact monolayer of the fully deprotonated BTB molecules covers the whole substrate surface (further referred to as compact δ-BTB layer) and is easily achievable. The compact δ-BTB layer is stable against mixing with pentacene, HAT-CN, and HM-TP, typical examples of organic semiconductors: we will show a thermodynamic preference for the formation of pentacene–BTB mixed phases for submonolayer coverages and demonstrate the robustness of the compact δ-BTB layer against structural and chemical changes. Our DFT calculations reveal that the compact δ-BTB layer possesses the lowest energy with respect to other possibilities, so they are robust from the thermodynamic point of view under UHV conditions.

### Photoelectron Spectroscopy

Aromatic carboxylic acids deprotonate (i.e., lose hydrogen from carboxylic–COOH groups) upon contact with metal substrates (except for gold) under UHV conditions.^51^ This chemical reaction occurs below room temperature for most metals, including Cu.^52^ The reaction is kinetically restricted on Ag surfaces, and annealing at elevated temperatures (30–50 °C) is usually required to obtain partially deprotonated molecular phases within minutes.^48^ However, significantly higher temperatures (∼200 °C) are necessary to achieve complete deprotonation because stable molecular phases hinder the deprotonation reaction.^48^ We have followed the deprotonation of BTB on both Ag(111) and Ag(100) substrates by photoelectron spectroscopy employing synchrotron radiation.

**Figure 2:**
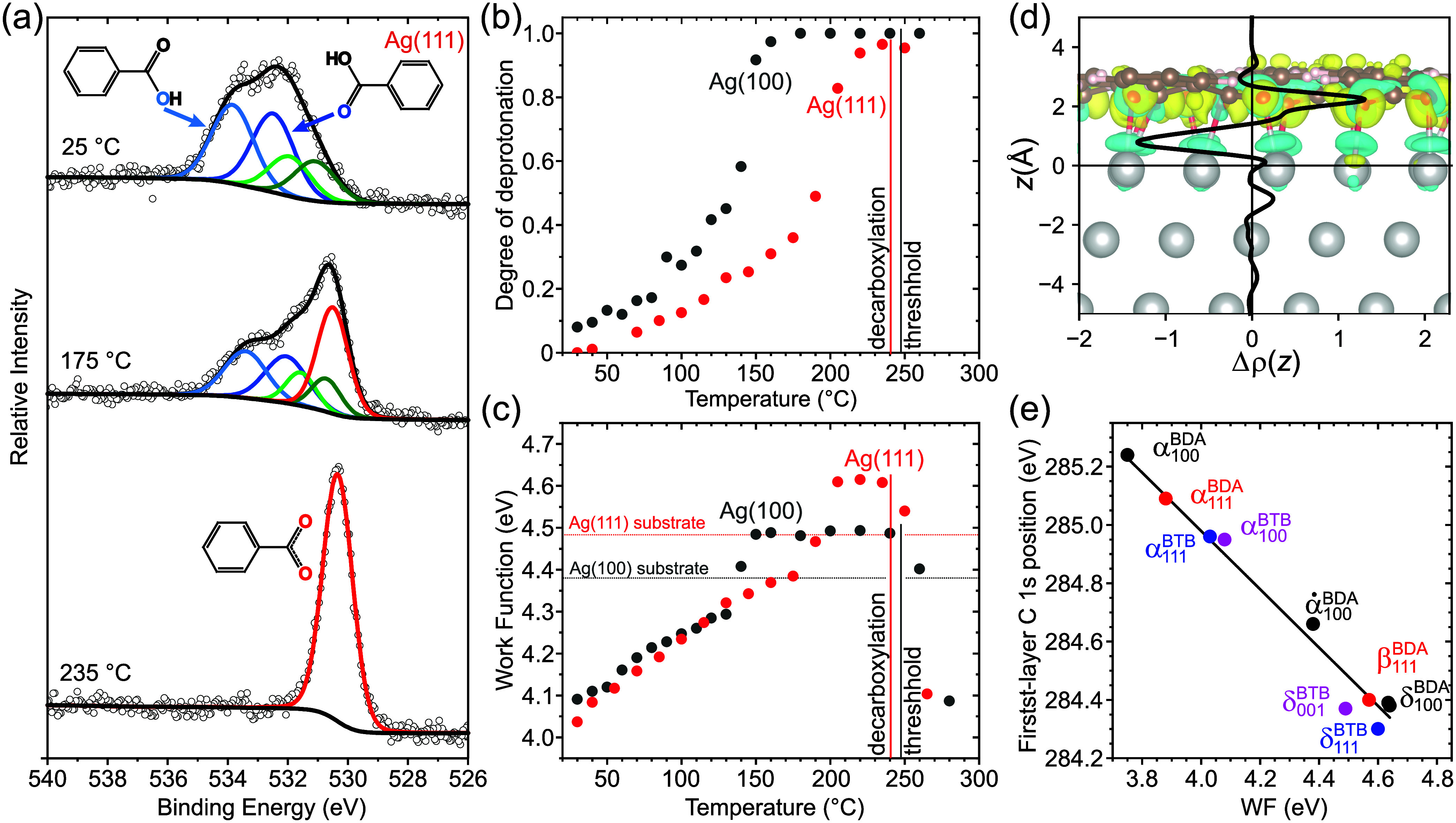
Changes in the electronic properties of BTB/Ag(111) during its gradual deprotonation. (a) Examples of O 1s spectra recorded on the as-deposited phase at 25 °C, after annealing at 175 °C, and annealing at 235 °C. (b) Degree of deprotonation of BTB carboxylic groups as a function of annealing temperature for both Ag(111) and Ag(100) surfaces. The vertical lines mark the threshold for decarboxylation, beyond which the decrease of O 1s peak intensity and decrease in sample WF is observed. (c) Sample WF as a function of annealing temperature for both Ag(111) and Ag(100) surfaces. The vertical lines mark the decarboxylation threshold; the horizontal lines mark the measured WF of the bare substrate surface. (d) Plane-averaged difference in charge density along the *z-*direction perpendicular to the δ-BTB/Ag(111) interface. The relaxed structure and the 3D isosurface of the charge density difference are depicted in the background. Silver, carbon, oxygen, and hydrogen atoms are in gray, brown, red, and white, respectively; electron depletion is colored blue, and accumulation yellow. (e) Position of the C 1s peak associated with phenyl rings within the first BTB molecular layer plotted as a function of the sample WF compared with earlier results for BDA.^34^ The line has a slope of −1, whereas the fitted experimental values have a slope of −1.03 ± 0.06.

The O 1s spectrum of 1 monolayer (ML) of as-deposited BTB molecules on Ag(111) shown in Figure 2a can be fitted by two pairs of peaks (light blue and blue; light green and green). As detailed in Supporting Information Section 1, we assign these peak components to carboxyl groups in two distinct binding motives. The intensity ratio of these pairs is 2:1. The higher binding energy component from each pair (highlighted by a lighter color in Figure 2a) is associated with hydroxyl oxygen (C–OH) and the darker one with carbonyl oxygen (−C=O) of the carboxyl group (−COOH) by comparison with previous works.^45,47,48^ Two distinct pairs of peaks point to the existence of two different chemical environments of the carboxyl groups; these are probably associated with the ribbon-like structure of the compressed as-deposited phase (see Figure S3 in Supporting Information Section 2).

During the annealing at progressively higher temperatures, a new component associated with carboxylate groups^45,47,48^ appears in the spectra and grows in intensity (red component in Figure 2a). The relative intensity of this peak is a measure of the degree of deprotonation of carboxylic groups (i.e., the fraction of deprotonated carboxyl groups with respect to all carboxyl groups) in the BTB layer. Figure 2b shows the evolution of the degree of deprotonation with annealing temperature for both Ag surfaces. On both surfaces, BTB molecules gradually deprotonate; on Ag(100), the deprotonation occurs at lower temperatures (consistently with BDA^48^), and complete deprotonation is observed at 170 °C, whereas on Ag(111), it is reached at 240 °C. For the Ag(111) substrate, this temperature is already very close to the threshold for the decarboxylation of BTB molecules, i.e., a complete removal of carboxyl groups that occurs around 250 °C for both surfaces. Above this threshold, the X-ray photoelectron spectroscopy (XPS) data show a decrease of oxygen-related signal, while the C 1s peak associated with phenyl rings keeps its intensity and shifts back to higher binding energies, i.e., 284.7 eV at Ag(111) and 284.9 eV at Ag(100), as the carboxylate-related dipoles cease to exist. Disordered polymer-like networks remain on the surface, as observed by STM (Figure S4 in Supporting Information Section 2). We observe (Figure 2b) that the fully deprotonated δ-BTB phase is stable in a broad window of temperatures of 170–250 °C on Ag(100) but only in a relatively narrow range of 235–250 °C on Ag(111).

The WF measured after each annealing is displayed in Figure 2c. The WF was determined from the position of the secondary electron cutoff.^34^ Due to the push-back effect, with increasing BTB coverage, the WF decreases below 4.1 eV on both surfaces.^14,34^ At higher temperatures, the gradual deprotonation leads to the formation of interfacial dipoles, and the WF increases again,^34^ reaching 4.61 eV on Ag(111) and 4.49 eV on Ag(100). A different WF of pristine surfaces explains this difference: the measured values were 4.38 and 4.48 eV for Ag(100) and Ag(111), respectively; their values are within the uncertainty interval of reported values, i.e., (4.36 ± 0.05) eV for Ag(100) and (4.53 ± 0.05) eV for Ag(111).^53^

To give a deeper insight into the adsorption-induced WF change, we characterized the structural and electronic properties of an δ-BTB/Ag(111) interface with ab initio calculations following the procedure described elsewhere.^34^ The change in the WF is attributed to the sum of the surface dipoles across the reorganized Ag substrate and the δ-BTB layer and the redistribution of the charge density at the interface resulting from molecule–substrate interaction. The smallest contribution of −0.06 D per BTB molecule arises from the substrate rearrangement. As shown in Figure 2d, subtle changes in the topmost silver layer give rise to this contribution. The intramolecular dipole moment caused by a bending of the molecule and shift of negatively charged oxygen atoms toward the substrate is calculated to be −2.89 D. Finally, the interface dipole moment calculated from plane-averaged charge density difference contributes with +3.57 D per BTB molecule. This contribution arises from a charge density difference plotted in Figure 2d, which shows electron depletion from the topmost silver layer and accumulation in the oxygen layer situated 2.2 Å from the substrate. The overall surface dipole density of the δ-BTB layer thus results in 0.62 D per BTB molecule, causing a 0.14 eV increase in WF from 4.49 eV for the pristine Ag(111) surface to 4.63 eV for the δ-BTB layer of Ag(111) surface in a perfect alignment with experimental observations.

In addition, we have measured the shift of energy levels for as-deposited (α-BTB) and fully deprotonated (δ-BTB) layers by analyzing the positions of phenyl-ring-related components of the C 1s peak for the first and second molecular layers; the procedure is described in our previous work.^34^ In Figure 2e, we have plotted the position of C 1s peak within the first layer for BTB together with values obtained for several BDA molecular phases obtained previously.^34^ The BTB data fit the previously established linear trend between the measured WF and core-level positions. The position of core levels experiences the same shift as the frontier orbitals in the case of vacuum level alignment.^31,54^

### STM and LEEM Investigation of δ-BTB Layers

STM and LEEM experiments have been carried out in our home UHV cluster system. We have explored submonolayer and full monolayer coverages of the fully deprotonated BTB phase (δ-BTB) on both Ag(111) and Ag(100) surfaces. As the results are very similar for both substrates, we will present only data for Ag(111) in the main text, and the data for the Ag(100) surface are given in Supporting Information Section 3.

To obtain the compact δ-BTB layer, the as-deposited BTB layers were annealed at temperatures necessary for the full deprotonation given in the previous section; the full deprotonation was proven by in situ XPS via the presence of a single O 1s peak component at 530.5 eV (Figure S9, Supporting Information Section 4), which is consistent with the synchrotron radiation data presented above. The structural evolution of molecular phases during gradual deprotonation was already described earlier in an STM work by Ruben et al.^55^ Our data of the as-deposited as well as partially deprotonated molecules are generally in line with their observations. In addition, we could reveal a high degree of complexity in the phase transformations in which the coverage and deposition rate play a significant role. However, a more detailed description of this is beyond the scope of this work.

**Figure 3:**
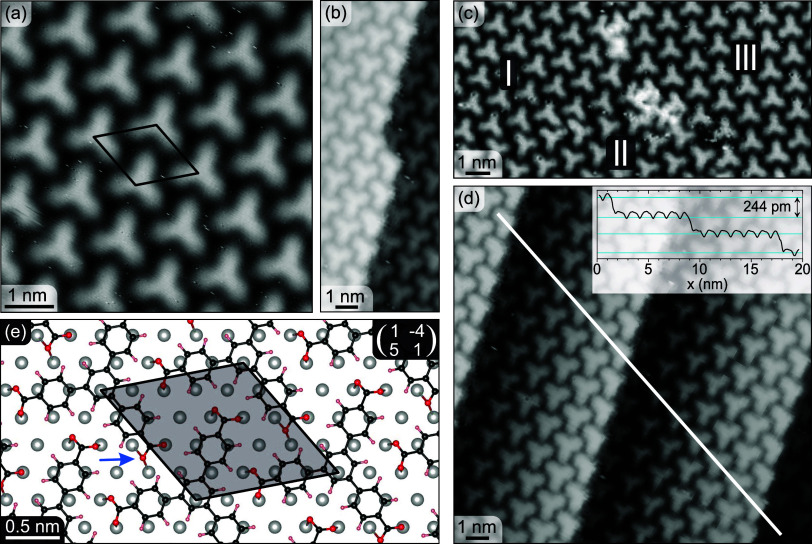
δ-BTB phase on Ag(111) surface. (a–d) Detailed STM images of the δ-BTB phase: (a) on a flat terrace showing the structure of the phase with the unit cell highlighted as a black rhombus; (b) growth of δ-BTB molecules across one step edge and an extended kink; (c) boundary of three δ-BTB domains marked I, II, and III (I and III are different rotational domains, whereas in I and II show a mirror symmetry); and (d) the δ-BTB phase with a single-orientation extending over several terraces; the inset shows a line scan along the white line indicated. Scanning parameters for all STM images: 1.4 V, 50 pA. The full-size images are given in Supporting Information Section 5. (e) DFT-based model of the δ-BTB phase showing the molecular arrangement on the Ag(111) surface: C: black, O: red, H: light red, Ag: gray. The highlighted unit cell is positioned in the same way as in (a); it features one molecule per unit cell and shows the adsorption positions of the three terminal carboxylate groups. Two carboxylates are aligned such that both O atoms adsorb in an on-top position. In the third carboxylate group, only one of the O atoms is in an on-top position, whereas the second is in a bridge position (highlighted by a blue arrow).

The molecular-scale topography of the δ-BTB phase obtained by STM shows the BTB molecules as bright protrusions of three-point stars in a hexagonally close-packed structure. The carboxylate (−COO) groups situated at the tips of the stars thereby point to the centers of neighboring molecules. This is shown in detail in Figure 3a, with the superstructure unit cell highlighted as a black rhombus. This phase was originally denoted as phase III with a degree of deprotonation of 2/3.^55^ However, our combined STM, XPS, and LEEM data clearly indicate that this phase is fully deprotonated. Figure 3b–d shows image details of the molecular structure on step edges and domain boundaries. Figure 3b shows the arrangement of the molecules along and over a single substrate step edge. All of the molecules at the upper side of the step edge show the same structure, with one point of the star protrusion missing. The arms of BTB molecules are partially flexible and thus can bend toward the lower terrace. This behavior is even more pronounced at a kink site where the BTB seems to have lost a complete arm. The kink also exactly follows the BTB shape and thus allows seamless growth of the compact δ-BTB layer over the step edge. In this way, the single domain extends over several monatomic steps, as shown in Figure 3d. This is evident from a line scan (see inset of Figure 3d) along the white line that shows a step height of ∼244 pm, which is slightly higher but in line with the step height of the Ag(111) substrate (236 pm). The molecular arrangement at the domain boundary is shown in Figure 3c. In our STM images, we have seen 4 orientations of molecules. In particular, we identify two different domain orientations (see Figure 3c, regions I and III) and two structural domains (I and II) that share the same unit cell but consist of molecules with orientation mirrored along the unit cell’s main diagonal. The calculated DFT model shown in Figure 3e is fully consistent with our STM data. It provides a deeper insight into the interface structure. BTB molecules are rotated by 10.5° with respect to the high-symmetry direction of Ag(111) substrate. The most common site for oxygen atoms to adsorb is in the on-top position, while one of the six oxygen atoms is situated in the bridge position (marked with a blue arrow).

**Figure 4:**
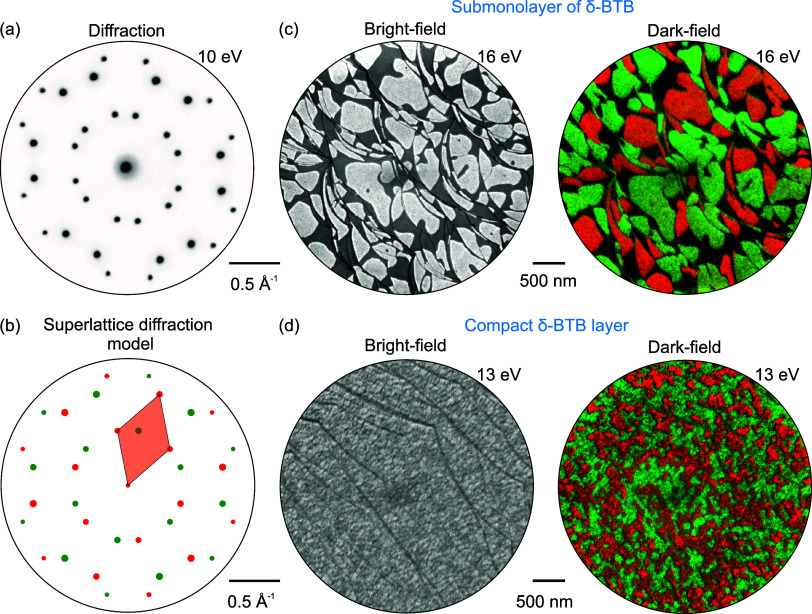
LEEM analysis of the δ-BTB phase on the Ag(111) surface. (a) Large-area diffraction pattern taken at 10 eV primary electron energy. (b) Superlattice diffraction model of the δ-BTB layer showing the composition from two single-domain diffraction patterns. (c) Bright- and dark-field images taken at the submonolayer BTB coverage showing δ-BTB islands; the green and red colors in the dark-field image are associated with a particular rotational domain given by the microdiffraction model in (b). (d) Bright- and dark-field images of the compact δ-BTB layer; the color coding is the same as in (c).

The LEEM measurements shown in Figure 4 provide real and reciprocal space views on sample morphology and structure at the mesoscale. The large-area diffraction pattern of the δ-BTB phase is presented in Figure 4a. The microdiffraction measurement reveals that the δ-BTB phase exists in two rotational domains on the Ag(111) surface: the model of the large-area diffraction pattern decomposed into two single-domain diffraction patterns is given in Figure 4b. The modeling of the δ-BTB diffraction pattern provides a  unit cell (in this work, all of the superstructure unit cells are given in the matrix notation). These two domain orientations were also identified in our STM images; see Figure 3c. In addition, each of these domains has an additional structural domain with the same unit cell but a mirrored orientation of molecules within them (see, e.g., Figure 3c). In the microdiffraction data and diffraction model, these two mirrored domains are indistinguishable.

The bright-field LEEM image (Figure 4c) portrays submonolayer coverage δ-BTB islands as a bright area on the dark background, which represents the bare substrate; the average area of the BTB islands is 0.3 ± 0.1 μm^2^. LEEM dark-field imaging, in which the image is formed only by electrons associated with a single diffraction spot different from the (0,0), allows real-space visualization of the rotational domains. For submonolayer coverage, individual δ-BTB islands grow in single-domain orientation. However, if the surface is completely covered (Figure 4d), we observe a larger number of smaller rotational domains within the δ-BTB layer; the upper bound of the average area of these domains is 0.011 ± 0.004 μm^2^, i.e., much smaller compared with the island size in the submonolayer coverage. The smaller domain size is probably caused by a limited BTB transport via surface diffusion, which is hindered in the full monolayer.^47^ Still, the δ-BTB surface shows a superior long-range order with a minimum of defects as the two domains are well matched at their boundary (see Figure 3c), and single domains extend across the step edges (see Figure 3b,d). On the other substrate facet, Ag(100), the structure of the compact δ-BTB layer is very similar to Ag(111) presented above: the molecular packing is the same with three BTB molecules per unit cell commensurate with the substrate and the area per molecule differs by 2% (see Supporting Information Section 6 for details).

We have tested the applicability of the compact δ-BTB layer as a CIL for OSs. In the following, we will describe the experiments with pentacene; the experiments with HAT-CN and HM-TP (Figure 1c–e) are given in Supporting Information Section 7.

### Formation of Mixed Pentacene–BTB Phases at Submonolayer BTB Coverage

**Figure 5:**
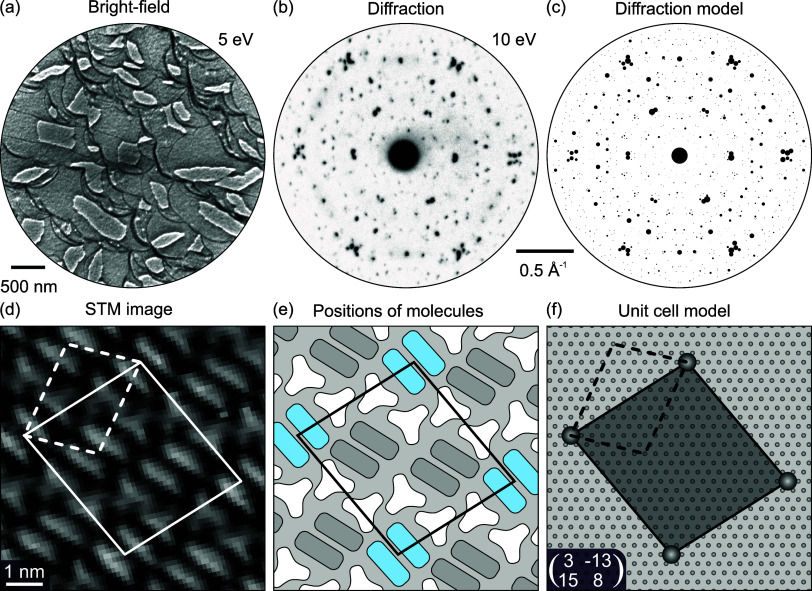
Pentacene–BTB mixed phase on Ag(111). (a) Bright-field image of the mixed phase formed by deposition of 0.5 ML pentacene and 0.5 ML BTB molecules and subsequent annealing at 170 °C. (b) Diffraction pattern originating from the mixed phase is shown in (a). (c) Diffraction model of the mixed phase shown in (b). (d) STM image of mixed pentacene–BTB phase with highlighted unit cell (solid line) and an apparent unit cell used for DFT calculations (dashed). (e) Schematics of arrangement of molecules within the unit cell obtained from STM. (f) Position of superstructure unit cell with respect to Ag(111) substrate.

At 1 ML coverage, δ-BTB molecules form a compact layer, which is stable against mixing with subsequently deposited organic semiconductor molecules. However, this changes in the submonolayer regime, where pentacene forms mixed phases with BTB. Deposition of 0.5 ML of pentacene and 0.5 ML BTB molecules on Ag(111) substrate and subsequent annealing (170 °C, 30 min) results in the formation of mixed pentacene–BTB phases. During the annealing, the BTB molecules deprotonate, and the pentacene–BTB mixed phases appear upon cooling. The bright-field image in Figure 5a shows molecular islands of the mixed phase (brighter areas) covering approximately 1/3 of the substrate, whose size and shape are restricted by the substrate step edges. The remaining molecules are present in molecular gas or disordered phases. The diffraction pattern (Figure 5b) measured on these islands is distinct from those observed for pure BTB phases. Employing ProLEED Studio to model the diffraction pattern (Figure 5c), we find the associated unit cell as . The STM analysis reveals that this phase comprises two close-laying pentacene molecules sandwiched between two δ-BTB molecules, as visualized in Figure 5d–f, giving the 1:1 ratio of pentacene and BTB. Moreover, the pair of pentacene molecules is tilted at the corners of the unit cell with respect to the two pentacene pairs in the interior, as shown in Figure 5d,e.

We note that the resulting molecular arrangement in mixed phases can be affected by the initial ratio of deposited molecules. In another experiment, we deposited 0.8 ML of pentacene BTB and 0.5 ML of BTB molecules and annealed the sample at 170 °C. After cooling, a wheel-like mixed phase with a 2:1 ratio was formed; see details in Supporting Information Section 8.

Mixed pentacene–BTB phases were formed in all experiments with a submonolayer coverage of BTB molecules. Mixed phases can be formed in several ways. One way is to deposit both molecules on the surface and obtain the mixture with subsequent annealing. Another possibility is to first create δ-BTB, deposit pentacene, and anneal the system afterward. The main parameters influencing the resulting structure for both procedures are the concentrations of both types of molecules on the surface and the annealing temperature, which needs to be high enough to reach the full deprotonation of the BTB molecules or dissolve δ-BTB islands but still below the decarboxylation and desorption onset.

These experiments with submonolayer BTB coverage indicate a thermodynamic preference for forming mixed molecular phases from pentacene and BTB over the separate pure molecular phases.

### Pentacene Deposition on the Compact δ-BTB Layer

**Figure 6:**
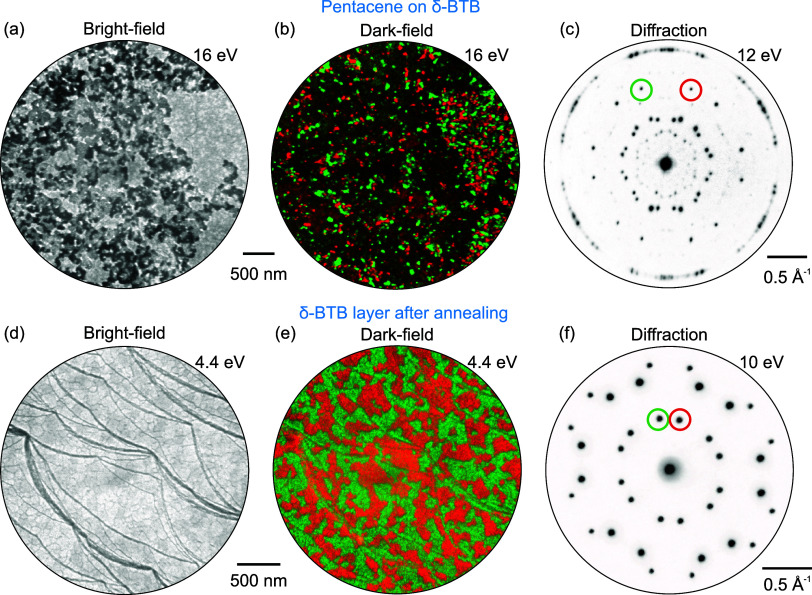
LEEM analysis of pentacene on compact δ-BTB layer on Ag(111). (a) LEEM bright-field image showing δ-BTB domains (brighter areas) partially covered by pentacene (darker areas). (b) Composition of dark-field images measured for the two δ-BTB orientational domains; the employed diffraction spots are marked in (c). Only areas without overlayer show a considerable intensity from the δ-BTB layer spots. (c) Diffraction pattern measured on pentacene deposited on the compact δ-BTB layer showing the sum of a faint pattern associated with δ-BTB and the one associated with the overlayer. (d, e) Bright- and dark-field images obtained after annealing show a compact δ-BTB layer present on the surface. (f) Diffraction pattern measured after annealing showing a bright δ-BTB pattern without any additional spots.

We have deposited pentacene on a sample covered by a compact δ-BTB layer. After the pentacene deposition, the LEEM bright-field image shows a compact δ-BTB layer covered with pentacene islands (Figure 6a) that appear as darker areas on a bright δ-BTB background. A LEEM dark-field analysis of δ-BTB spots given in Figure 6b reveals that BTB molecules still cover the whole surface, and the pentacene overlayer attenuates the δ-BTB signal. Figure 6c shows a diffraction pattern that is a superposition of a pronounced diffraction pattern associated with a crystalline overlayer, likely associated with pentacene, and a faint pattern associated with the δ-BTB layer located below.

**Figure 7:**
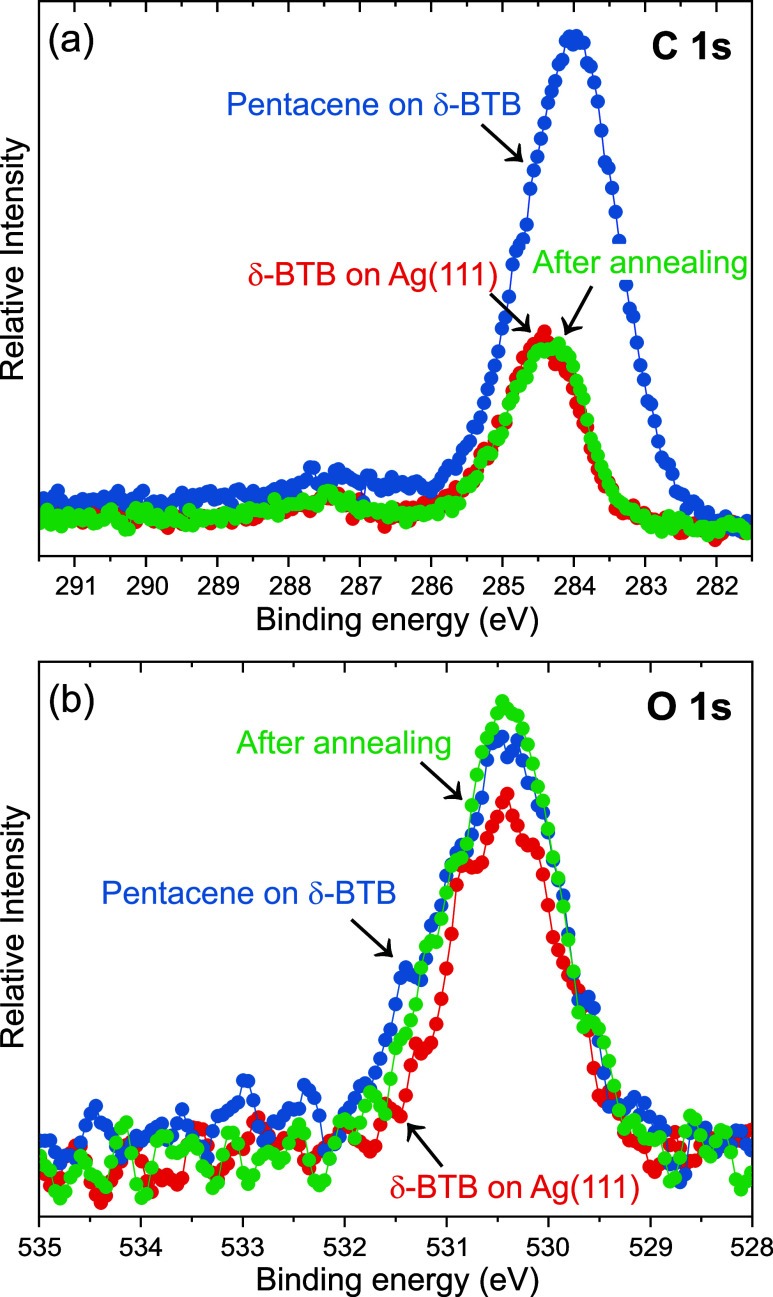
XPS analysis of pentacene on the compact δ-BTB layer on Ag(111). (a) C 1s and (b) O 1s spectra measured on the compact δ-BTB layer (red), after pentacene deposition (blue), and subsequent sample annealing at 100 °C (green).

Annealing the sample at 100 °C for 15 min induces the complete desorption of pentacene: the LEEM/low-energy electron diffraction (LEED) results (Figure 6d–f) show a compact δ-BTB layer similar to that before the pentacene deposition. We did not reveal any sign of the formation of mixed phases comprising BTB and pentacene. XPS spectra of C 1s and O 1s taken before (red) and after (blue) pentacene deposition and sample annealing (green) are given in Figure 7. After pentacene deposition, we observe an increase in the intensity of the C 1s peak, which decreases to the original one after annealing. The O 1s peak shows only a slight change both after deposition and annealing, as pentacene comprises only carbon atoms.

Based on XPS and LEEM observations, we conclude that the full δ-BTB layer is robust against the mixing with pentacene. This robustness can be either of thermodynamic or kinetic origin. The fact that pentacene and BTB form mixed phases suggests that forming bonds between pentacene and δ-BTB molecules is favorable, which indicates the kinetic origin of the robustness. However, the DFT analysis given below shows the opposite, as at coverages approaching a full monolayer, the adsorption energy per unit area dictates the thermodynamic stability of the compact δ-BTB layer.

### DFT Calculations: Thermodynamic Stability of the Intermixed Phase and δ-BTB Layer

In the following, we demonstrate the energetic preference of the mixed pentacene–BTB phase in the submonolayer coverage and the preference for the δ-BTB phase at full monolayer coverage. In both cases, the decisive factor that determines the stability is the adsorption energy of a molecule per unit area calculated as
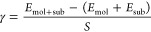
1where *E*_mol+sub_ is the total energy of a molecular phase on a substrate with area *S*, *E*_mol_ denotes gas-phase energies of δ-BTB and pentacene molecules, and *E*_sub_ is the total energy of a bare substrate (see Supporting Information Section 9 for the results if a protonated BTB in the gas phase is used as an energy reference). Monolayers of pentacene and δ-BTB were modeled with periodic boundary conditions using the Ag(111) supercells given by  and , respectively. Due to its size, the real superstructure unit cell for the pentacene–BTB mixed phase  is approximated by a smaller, apparent unit cell of  depicted in Figure 5f and in Figure S18c. This induces ∼3% strain in the shorter surface vector and 2.5% angular strain. Reference energies for the silver substrate were calculated for each supercell separately. The resulting stabilities, i.e., absolute adsorption energies and energies per unit area, for pentacene, δ-BTB, and the mixed phase on Ag(111) substrate are summarized in Table 1. We note that, in line with experiments, our DFT calculations do not show any surface reorganization, which is not favored due to a relatively strong intermolecular interaction, which hinders the lifting of Ag atoms out of the normal Ag(111) plane. This conclusion is further supported by our benchmark calculations involving fully deprotonated trimesic acid (TMA), which lacks attractive intermolecular interactions. In the case of TMA, silver atoms with three Ag–O bonds were lifted up, in line with previous works showing silver clusters in the molecular layer.^56^ However, the diffraction model of the δ-BTB layer excludes such scenarios due to steric reasons: in the case of BTB, carboxyl groups are too far away to form 3-fold Ag sites, and the molecular unit cell is too small to accommodate any silver adatom/cluster.

**Table 1:** Calculated Adsorption Energies Per Molecule (*E*_ads_) and Energies Per Unit Area (γ) for Pentacene, Deprotonated BTB (δ-BTB), and Intermixed Pentacene–BTB Layer, Using PBE-D3 and optB86 Functionals^a^

	*E*_ads_ (eV)		γ (meV/Å^2^)	
molecular layer	PBE-D3	optB86b	PBE-D3	optB86b
pentacene	–2.60	–2.35	–20.0	–18.0
δ-BTB	–9.36	–9.44	–61.7	–62.0
intermixed (from exp. diffraction)	–12.05^b^	–11.79^b^	–42.8	–41.7
intermixed (most stable)	–12.14^b^	–11.94^b^	–47.3	–46.4

a *E*_ads_ for the intermixed phases is given for a pair comprising one BTB and one pentacene molecule, giving higher stability than pure molecular counterparts, i.e., a sum of the first two rows in a column.

b Per pentacene–BTB pair.

First, we will evaluate the preferred molecular phase in the case of the fully covered surface. There are two main contributions that decrease the free energy of the system: molecule–substrate bonding and intermolecular bonding. The computed energies per unit area reveal that the δ-BTB layer has by ∼15 meV/Å^2^ lower free energy per unit area than the mixed phase, i.e., the δ-BTB layer is more stable. This energy preference is elucidated by relatively strong Ag–O bonds, with a calculated binding energy of −1.7 eV, and supplemented by the contribution of attractive intermolecular interactions that stabilize the δ-BTB structure by an additional 0.8 eV per molecule. The strong attachment to the substrate results in the preference of BTB adsorption over the physisorbed pentacene. Hence, the complete δ-BTB layer shows a weak thermodynamic preference over the mixed phase.

Now, we will address the submonolayer coverages. The decisive parameter is still the surface free energy per unit area. However, in this case, there is a free substrate to accommodate all of the adsorbed molecules irrespective of their bonding strength to the substrate. Since we are not restricted to the available surface area, the energy per molecule can be used to assess the preference for forming either pure or mixed phases. Our results show that the total adsorption energy per pentacene–BTB pair is 90 meV (PBE-D3) or 10 meV (optB86b) lower for the intermixed phase compared to the separate phases. However, the calculated stability is affected by imposed strain and the restriction to periodically repeating molecules that retain energetically unfavorable positions. To assess the validity of the results for the mixed structure, we have also computed its stability using modified supercells of similar dimensions but with different orientations with respect to the substrate, as shown in Supporting Information Section 10. In this case, the highest stability achieved favors the mixed phase by 180 meV (PBE-D3) and 150 meV (optB86b) per one pentacene–BTB pair. These values present a lower limit for the stability of the mixed phase compared to the separate counterparts. In summary, these results point to the thermodynamic stability of the pentacene–BTB mixed phase for submonolayer coverages, which is consistent with experimental observations.

In the next step, we evaluate the kinetic barrier for breaking the compact δ-BTB layer. Due to the robust Ag–O bonds linking the BTB molecules to the silver substrate, the most likely scenario of disrupting the δ-BTB layer is to reprotonate the carboxyl groups, thus weakening their bonds to the surface, allowing their subsequent detachment from the surface. The deprotonated state is favored for a flat-laying BTB molecule, whereas the protonated carboxyl group is preferred for the BTB molecule detached from the surface. In detail, for a detached BTB, there is a 1.8 eV free energy preference for the protonated carboxyl group compared with the deprotonated group and 1/2 of H_2_ molecule, taking into account the chemical potential of molecular hydrogen under conditions routinely reached during our experiments (−1.07 eV at 25 °C, 2 × 10^–10^ mbar). On the contrary, for the flat-laying BTB molecule, the formation of the O–H bond from molecular hydrogen is not favored; the free energy is by 0.2 eV higher compared with the molecular hydrogen under UHV conditions as the proximity of the silver substrate weakens the O–H bond. Therefore, the most probable way to disrupt the δ-BTB layer involves reprotonation of one of the carboxylic groups and its separation from the surface, resulting in a standing-up BTB configuration with the other two carboxylate groups attached to the substrate.

**Figure 8:**
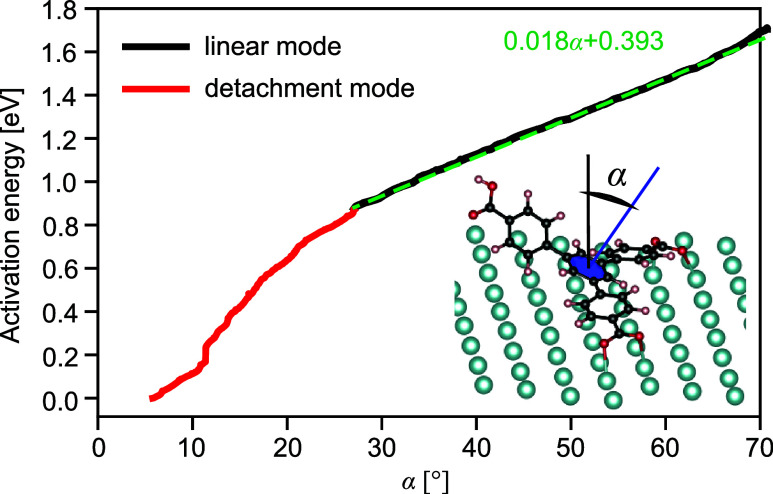
Detachment of one singly protonated BTB molecule from the δ-BTB layer. For clarity, only the molecule being detached is shown. The detachment process is described as a function of the angle α between the *z*-axis and a normal vector of the plane that intersects the central phenyl ring (marked as blue in the inset). This process is composed of a nonlinear mode up to 27° and 0.87 eV (red line). Above 27°, the trend is linear up to 70° with an energy step of 18 meV per 1° (black line).

To estimate the energy barrier for opening the compact δ-BTB layer, one BTB molecule in the 2 × 2 supercell was arranged in the standing-up configuration, the lifted carboxylate group was protonated by additional hydrogen, and the whole structure was allowed to relax back to the flat-lying configuration. Figure 8 shows this process as a function of angle α between the *z*-axis and a normal vector of the plane, which intersects the central phenyl ring. The detachment is composed of two modes: First, the nonlinear up to 27° and the total energy difference between two limiting configurations of 0.87 eV; within this interval, the attractive intermolecular and molecule–substrate interactions are broken. The second mode shows a linear trend with an energy step of 18 meV per 1°. This behavior holds up to 70°, in which the total energy difference is estimated to be 1.7 eV. Initial and final structures are provided in Supporting Information Section 10. On the Ag(111) surface, the activation energy for the dissociation of hydrogen molecules amounts to 1.3 eV,^57^ which is significantly larger than the barrier of 0.87 eV for the layer opening. This makes the hydrogen dissociation the rate-limiting step and the δ-BTB layer also kinetically stable at room temperature.

### Discussion of the Origin of the Robustness of the Compact δ-BTB Layer

Our experimental data and DFT calculations show the thermodynamic preference for the formation of mixed δ-BTB–pentacene phases. However, at the full coverage, the δ-BTB layer becomes preferred. This seemingly contradictory statement comes from the strong binding of carboxylate groups to the silver substrate, which defines the molecular layer structure. Hence, the other effects can take place only if all BTB molecules are bound to the substrate. Thus, for submonolayer coverages, there is a free area to satisfy the stability condition for the formation of the mixed pentacene–BTB phases, which are formed in the presence of supercritical^58^ pentacene concentration.

The compact δ-BTB layer can be obtained by depositing >1 ML of BTB and subsequent sample annealing at the specific temperature. The excessive BTB desorbs from the surface, resulting in a compact δ-BTB layer without remaining BTB in the second layer. In contrast, obtaining the full layer of the BDA molecules (previous studies) was challenging as they display significant desorption from the first layer at temperatures close to full deprotonation. On Ag(111), the maximum coverage of the fully deprotonated BDA phase was around 50%, and on Ag(100), it was between 90 and 95%.

In the formation of the compact layer of deprotonated carboxylic acid molecules, the capability of filling the residual open sites is essential. This can be done by filling the gaps with molecules from the second layer. In the case of BTB, there are enough molecules in the second layer as their desorption temperature from the second BTB layer is close to the temperature required for their full deprotonation. On the contrary, the BDA molecules desorb from the second layer between 75 and 100 °C,^34^ i.e., ∼100 °C below the temperature required for the full deprotonation (180–200 °C).^48^ Hence, in the BDA case, there are no BDA molecules left to fill the gaps formed during the formation of the fully deprotonated phase.

The other essential feature is eliminating weak points in the layer that are generally localized at domain boundaries and step edges. Our STM images clearly show a favorable matching of molecular domains at their boundary so that they show minimum defects. The alignment of molecules at the step edges is generally challenging. However, the δ-BTB phase extends over several terraces, seamlessly growing over the step edges. The structure of BTB at the step edges is uniform along them, including the special shape of kink sites. The only possibility to achieve the perfect alignment of step edges with the growing molecular phase is to adjust their morphology to fit the growing molecular phase. The extended growth of 1D molecular chains,^59,60^ 2D molecular phases,^61,62^ or 2D metal–organic frameworks^63^ featuring carboxylic^59−61,63^ or cyano^62^ groups was reported. This behavior was ascribed to the robust intermolecular bonding,^59,60,62^ flexibility of the molecular backbone,^59,60^ and adaptation of the substrate-step-edge shape and the terrace widths.^61,63^ In our case, two contributions, i.e., molecular flexibility and changing the shape of the step edges, are important. Reshaping the substrate thus should be taken as an inherent part of the self-assembly of δ-BTB molecules, essential for the growth of the compact δ-BTB layer.

## Conclusions

In this paper, we have introduced the robust dipolar layers based on the deprotonated tricarboxylic acid polyphenylene molecule, δ-BTB. The δ-BTB layer prepared on both low-energy facets of silver, Ag(111) and Ag(100), covers the entire sample surface. This is enabled by the active sculpting of the surface by the BTB molecule, i.e., adjusting the position and shape of the step edges to fit the molecular layer precisely to the terraces and enable its seamless growth over the step edges. Similarly, the BTB molecules show a favorable matching at domain boundaries, which display a very low number of defects. The layer possesses the out-of-plane electric dipole that shifts the energy level of molecular layers deposited above, e.g., organic semiconductors, and, thus, can be employed as a monolayer thick charge injection layer for efficient charge injection from a metal electrode to an organic semiconductor film.

We have demonstrated the robustness of the layer toward organic semiconductors deposited on top of the compact δ-BTB layer and subsequent annealing at temperatures higher than typical processing temperatures of OS devices. The stability of the δ-BTB layer is given by the fact that it represents the energetically most favorable configuration. Even if one of the groups was protonated (e.g., under operational conditions), there still would be a considerable kinetic barrier associated with the disruption of the layer that would result in a possible loss of functionality.

Our work thus provides a robust molecular layer that can be employed as a wetting layer of subsequent growth of functional molecular films while it allows the control of substrate work function by adjusting the dipole density via the design of the molecular precursor. Our theoretical insights define the design criteria that ensure the robustness of the layer and allow the engineering of the layers with tailored functionality.

## Methods

The experiments, including STM, LEEM, and XPS, were carried out in a complex ultrahigh vacuum (UHV) system at the CEITEC Nano Research Infrastructure, CZ. The system comprises several UHV chambers connected via a UHV transfer line. The investigated samples were prepared and analyzed in distinct UHV chambers, between which the samples were transferred through a UHV transfer line (base pressure 2 × 10^–10^ mbar). During the transfers (60–150 s), the pressure increased to ∼2 × 10^–9^ mbar but quickly recovered to the base level when the movement ceased.

### Sample Preparation

The Ag(111) and Ag(100) single crystals (SPL) were cleaned by repeated cycles of Ar^+^ sputtering and annealing at 520 °C, followed by slow cooling (<1 K/s) to room temperature in the “preparation chamber” (base pressure of 2 × 10^–10^ mbar). BTB molecules were subsequently deposited in an adjacent “deposition chamber” either by (i) a near-ambient effusion cell (Createc) from an oil-heated crucible held at 208 °C or (ii) a Standard Effusion Cell (WEZ40, MBE Komponenten) from a resistively heated alumina crucible also held at 208 °C on the sample held at room temperature. Pentacene, HM-TP, and HAT-CN molecules were all deposited from an Organic Material Evaporator Quad Cell (OEZ40, MBE Komponenten) from a resistively heated quartz crucible at temperatures 173 °C for pentacene, 170 °C for HM-TP, and 217 °C for HAT-CN on the samples held at room temperature. All mentioned molecular powders were purchased from Sigma–Aldrich (>97% purity) and deposited after thorough degassing under UHV conditions at a pressure lower than 5 × 10^–9^ mbar. The complete monolayer (ML) of BTB was obtained after 30 min deposition. We define 1 ML of BTB as a completely covered surface with the fully deprotonated phase, i.e., 0.045 BTB molecules per substrate unit cell area for Ag(111) and 0.048 for Ag(100). One ML of pentacene is defined as full coverage of pentacene forming p(6 × 3) superstructure on Ag(111) surface,^64^ i.e., 0.056 pentacene molecule per substrate atom.

### Sample Analysis

Scanning tunneling microscopy images were recorded with a commercial system Aarhus 150 (Specs) equipped with a KolibriSensor and a base pressure of 1 × 10^–10^ mbar. Images from the STM measurements were acquired at room temperature in constant current mode; the sample bias voltage was set between 1.0 and 1.4 V, and the tunneling current was set to 50 pA, if not otherwise indicated in the images. The STM images (and diffraction patterns, see LEEM below) were rotated to align the horizontal direction of the images with the main crystallographic direction of the Ag(111) substrate. The drift distortion in the images was corrected by transforming the image to match the unit cell determined in STM with the one obtained from diffraction in LEEM. LEEM/LEED experiments were performed in a Specs FE-LEEM P90 instrument with a base pressure of 2 × 10^–10^ mbar. Bright-field images were formed by electrons collected from the (0,0) diffracted beam. Diffraction patterns were obtained from a 15 × 10 μm^2^ surface area, and microdiffraction was done with a 185 nm e-beam spot size on the sample. The diffraction patterns were modeled using ProLEED Studio;^65^ a local congruence approach^45,46^ was employed to match the modeled diffraction patterns with the measured ones. XPS was performed on a Specs system utilizing a Phoibos 150 spectrometer. All measurements employed a nonmonochromatized X-ray source with Mg Kα radiation (*h*ν = 1253.6 eV), emission angle 0°, and the high-magnification mode with the iris aperture set to 15 mm. Detailed spectra were acquired in medium magnification mode, 20 eV pass energy, 0.1 eV energy step for C 1s and 0.05 eV for O 1s region; for each spectrum 40 sweeps with 0.1 s dwell time were summed. The total resolution (accounting for analyzer and excitation radiation contributions) was 800 meV. The photoelectron spectra were subsequently fitted by Voigt profiles after a Shirley or (Shirley + linear) background subtraction. Synchrotron radiation photoelectron spectroscopy was performed at the Materials Science Beamline at the Elettra synchrotron light source in Trieste, IT. We used excitation energies of 420, 510, and 670 eV for the C 1s, Ag 3d_5/2_, and O 1s peaks, respectively. Detailed spectra were acquired in medium area lens mode using 10 eV pass energy integrating 2 (Ag 3d), 5 (C 1s), or 20 (O 1s) sweeps; the dwell time was set to 0.1 s, and the energy step size to 0.05 eV. The total resolution was in the range of 300–550 meV. Peak positions were corrected with respect to the measured Fermi edge of the Ag substrate and intensity for the photocurrent of a gold mesh placed in the beamline. The temperature was read by a K-type thermocouple attached to the bottom side of the sample plate (Ta, thickness 0.1 mm). The WF was determined from the position of the secondary electron cutoff as described earlier.^34^

### Computational Details

All density functional theory calculations are carried out using the Vienna Ab initio Simulation Package (VASP).^66^ For silver, carbon, hydrogen, and oxygen, 11 valence electrons, 4 valence electrons, 1 valence electron, and 6 valence electrons, respectively, are described with a plane-wave basis set with an energy cutoff set to 450 eV, while all core electrons are treated by the projector augmented wave method (PAW).^67^ The exchange–correlation energy is described by the PBE-D3 functional^68,69^ and benchmarked with the nonlocal optB86b van der Waals functional,^70^ which shows no qualitative differences in the presented results. Structural and electronic calculations are performed in two steps: first, for all structural relaxations, we use a sparser *k*-points grid: 3 × 3 × 1 γ-centered Monkhorst–Pack grid^71^ for the δ-BTB phase and γ-only calculations for other structures; and the geometry optimization is stopped when all residual forces acting on ions are smaller than 0.01 eV/Å. Second, electronic structure calculations are performed as single-point calculations starting from optimized structures with a denser sampling of the Brillouin zone with a grid density larger than 60 *k*-points per Å^–1^. The Ag(111) surface is modeled with a 5-layered slab, and all calculations account for dipole corrections to the potential and energy.

## Data Availability

The data that support the findings of this study are available from the corresponding author upon reasonable request.

## Supporting Information Available

Synchrotron radiation photoelectron spectroscopy analysis of the BTB deprotonation on Ag(111) and Ag(100); STM images of the as-deposited phase and decarboxylated polymeric network; compact δ-BTB layer and pentacene–BTB mixed phases on Ag(100); laboratory XPS analysis of the compact δ-BTB layer; supporting STM figures of δ-BTB on Ag(111); comparison of δ-BTB unit cell on Ag(100) and Ag(111); stable overlayers of HM-TP and HAT-CN; wheel-like pentacene–BTB mixed phase on Ag(111); gas-phase reference for calculated adsorption energies; and supporting DFT structures of molecular monolayers on Ag(111) substrate (PDF)
