# Supplementary material for: Robust Dipolar Layers between Organic Semiconductors and Silver for Energy-Level Alignment
Source: ACS Appl Mater Interfaces. 2024 Mar 29;16(14):18099–111. doi: 10.1021/acsami.3c18697 (PMC11009919; doi:10.1021/acsami.3c18697)
Supplement: Supplementary file 1 — am3c18697_si_001.pdf [file am3c18697_si_001.pdf]

SUPPORTING INFORMATION

# Robust Dipolar Layers between Organic Semiconductors and Silver for Energy-Level Alignment

*Tomáš Krajňák,[1] Veronika Stará,[1] Pavel Procházka,[1] Jakub Planer,[1] Tomáš Skála,[2] Matthias Blatnik,[1] Jan Čechal[1,3]**

[1] CEITEC – Central European Institute of Technology, Brno University of Technology, Purkyňova 123, 612 00 Brno, Czech Republic.

[2] Department of Surface and Plasma Science, Faculty of Mathematics and Physics, Charles University, V Holešovičkách 2, 180 00 Prague 8, Czech Republic.

[3] Institute of Physical Engineering, Brno University of Technology, Technická 2896/2, 616 69 Brno, Czech Republic.

* E-mail: cechal@fme.vutbr.cz (J. Č.)

CONTENTS:

1. Synchrotron radiation photoelectron spectroscopy analysis of the BTB deprotonation on Ag(111) and Ag(100).

2. STM images of the as-deposited phase and decarboxylated polymeric network.

3. Compact δ-BTB layer and pentacene–BTB mixed phases on Ag(100).

4. Laboratory XPS analysis of the compact δ-BTB layer.

5. Supplementary STM figures of δ-BTB on Ag(111).

6. Comparison of δ-BTB unit cell on Ag(100) and Ag(111).

7. Stable overlayers of HM-TP and HAT-CN.

8. The wheel-like pentacene-BTB mixed phase on Ag(111).

9. Gas-phase reference for calculated adsorption energies.

10. Supplementary DFT structures of molecular monolayers on Ag(111) substrate.

## 1. Synchrotron radiation photoelectron spectroscopy analysis of the BTB deprotonation on Ag(111) and Ag(100)

Contrary to our previous papers, where BDA molecules were employed, we could not unambiguously relate the measured spectra to well-defined molecular phases measured by LEEM. Hence, the XPS analysis is targeted primarily to obtain the degree of deprotonation at given annealing temperatures and relate it to simultaneously measured work function.

The C 1s spectra were measured after annealing up to a temperature of 280 °C. An example of spectra measured for as-deposited, intermediate, and fully deprotonated BTB phases is given in Figure S1 for both Ag(111) and Ag(100) substrates. The main component of C 1s spectra at ~285 eV can be associated with carbon atoms in phenyl rings and the small component at higher binding energies (287 – 289 eV) with carboxylic carbon. The fitting of the C 1s spectra is not straightforward due to the presence of shake-up satellites; however, detailed fits are unnecessary for this work. Hence, the C 1s spectra are presented without fitting in Figure S1.

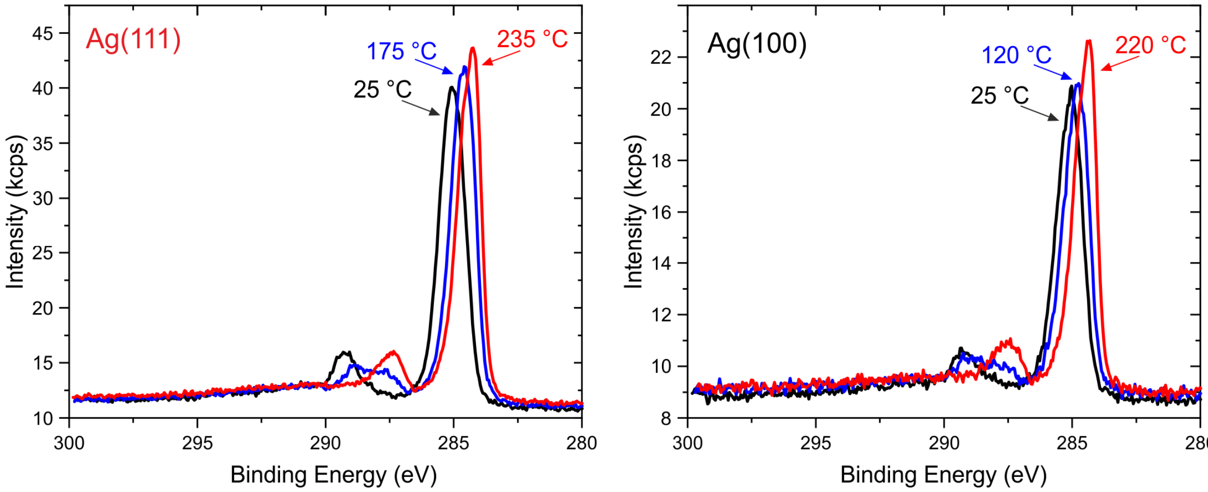


**Figure S1:** C 1s spectra of BTB layer measured by synchrotron radiation photoelectron spectroscopy for as-deposited sample (black), after annealing at the temperatures of 175 or 120 °C (blue), and 235 or 220 °C (red) on Ag(111) and Ag(100) surfaces, respectively.

## Fitting of the O 1s spectra

The O 1s spectra were fitted by up to 5 Voigt components and a Shirley background, as shown in Figure S2; the peak fitting parameters are summarized in Tables S1 for Ag(111) and S2 for Ag(100). The O 1s spectrum of the as-deposited BTB can be fitted by two pairs of peaks: the first pair (O1) at 533.8 + 532.5 eV (light blue and blue in Figure S2) and the second pair (O2) at 532.0 + 531.1 eV (light green and green in Figure S2). The intensity ratio of these pairs was 2:1. Within the pair, the intensities of the components have a ratio of 1:1; the higher binding energy component can be associated with hydroxyl, and the lower binding energy component with carbonyl oxygen. For annealed samples, we have added a peak component (O3) associated with a deprotonated carboxyl group (red in Figure S2); a single component is due to the symmetric chemical state of both oxygen atoms. The degree of deprotonation of carboxyl groups was determined as a ratio of carboxylate (O3) to the total intensity of O 1s peak. For BDA (previous works), we introduced two additional components related to the intact carboxyl group bound to the deprotonated one; their position would be similar to the green components (O2). Therefore, in the analysis of BTB, we did not include them, which results in a generally imprecise fitting, which, however, gives the correct degree of deprotonation.

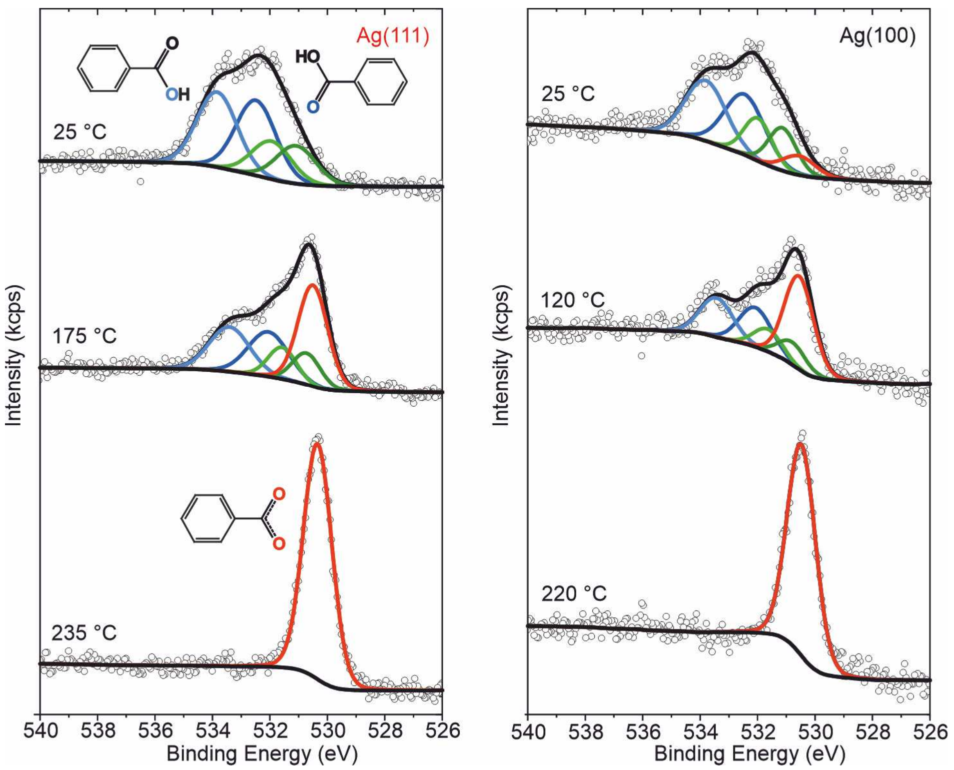


**Figure S2**: Example of fitting of the O 1s spectra measured by synchrotron radiation photoelectron spectroscopy for as-deposited sample (top row), partially deprotonated BTB annealed at the temperature of 175 or 120 °C (middle), and fully deprotonated BTB annealed at 235 or 220 °C (bottom) on Ag(111) and Ag(100) surfaces.

**Table S1:** Peak-fitting parameters of the O 1s peak for BTB deposited on **Ag(111) substrate.** Peak binding energy (BE) and FWHM of the Gaussian part are given for the as-deposited BTB layer (protonated), BTB annealed at 175 °C, and annealed at 235 °C (fully deprotonated). Voigt function was used for fitting; the width of the Lorentzian component was 0.1 eV.

| | **Components O1** | | **Components O2** | | **Component O3 (carboxylate oxygen)** | |
|---|---|---|---|---|---|---|
| | BE (eV) | FWHM (eV) | BE (eV) | FWHM (eV) | BE (eV) | FWHM (eV) |
| **Deposited** | 533.84 + 532.49 | 1.6 | 531.94 + 531.09 | 1.6 | – | – |
| **Intermediate** | 533.40 + 532.05 | 1.6 | 531.57 + 530.72 | 1.1 | 530.50 | 1.2 |
| **Deprotonated** | | | | | 530.33 | 1.1 |

Positions of Ag $3d_{5/2}$ peak were in the interval of (368.23 ± 0.01) eV for all the measurements.

**Table S2:** Peak-fitting parameters of the O 1s peak for BTB deposited on **Ag(100) substrate.** Peak binding energy (BE) and FWHM of the Gaussian part are given for the as-deposited BTB layer (protonated), BTB annealed at 120 °C, and annealed at 220 °C (fully deprotonated). Voigt function was used for fitting; the width of the Lorentzian component was 0.1 eV.

| | **Components O1** | | **Components O2** | | **Component O3 (carboxylate oxygen)** | |
|---|---|---|---|---|---|---|
| | BE (eV) | FWHM (eV) | BE (eV) | FWHM (eV) | BE (eV) | FWHM (eV) |
| **Deposited** | 533.78 + 532.45 | 1.6 | 531.98 + 531.13 | 1.1 | 530.55 | 1.3 |
| **Intermediate** | 533.42 + 532.07 | 1.3 | 531.64 + 530.79 | 1.1 | 530.55 | 1.1 |
| **Deprotonated** | | | | | 530.51 | 1.1 |

Positions of Ag $3d_{5/2}$ peak were in the interval of (368.20 ± 0.01) eV for all the measurements.

## 2. STM images of the as-deposited phase and decarboxylated polymeric network

Figure S3 shows the STM image of the as-deposited BTB phase on Ag(111), in which BTB molecules form a compressed ribbon-like structure. A polymeric network of decarboxylated molecules formed after sample annealing at 250 °C is shown in Figure S4.

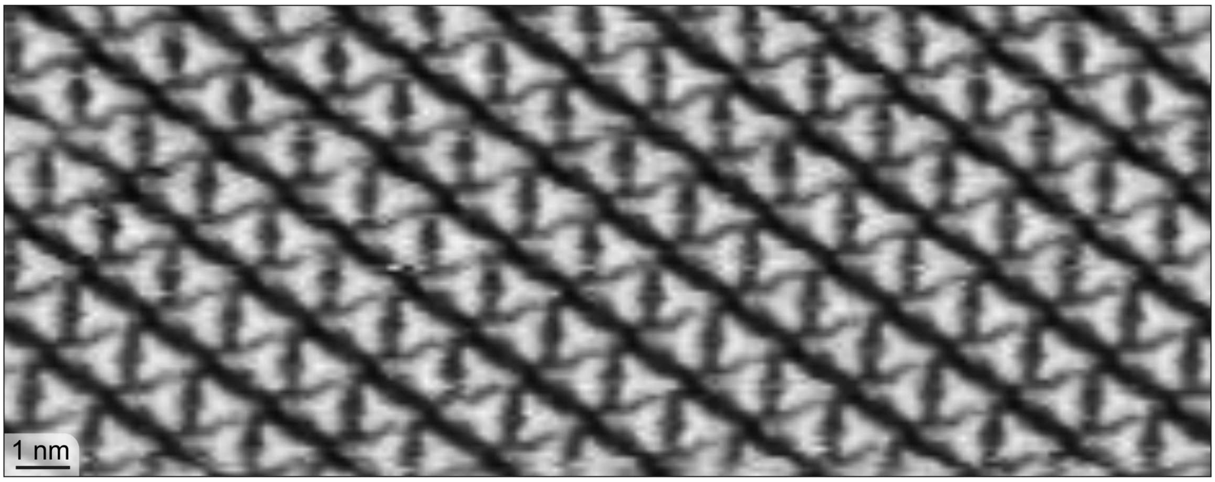


**Figure S3:** As-deposited BTB phase on Ag(111). Scanning parameters: 1.2 V, 50 pA.

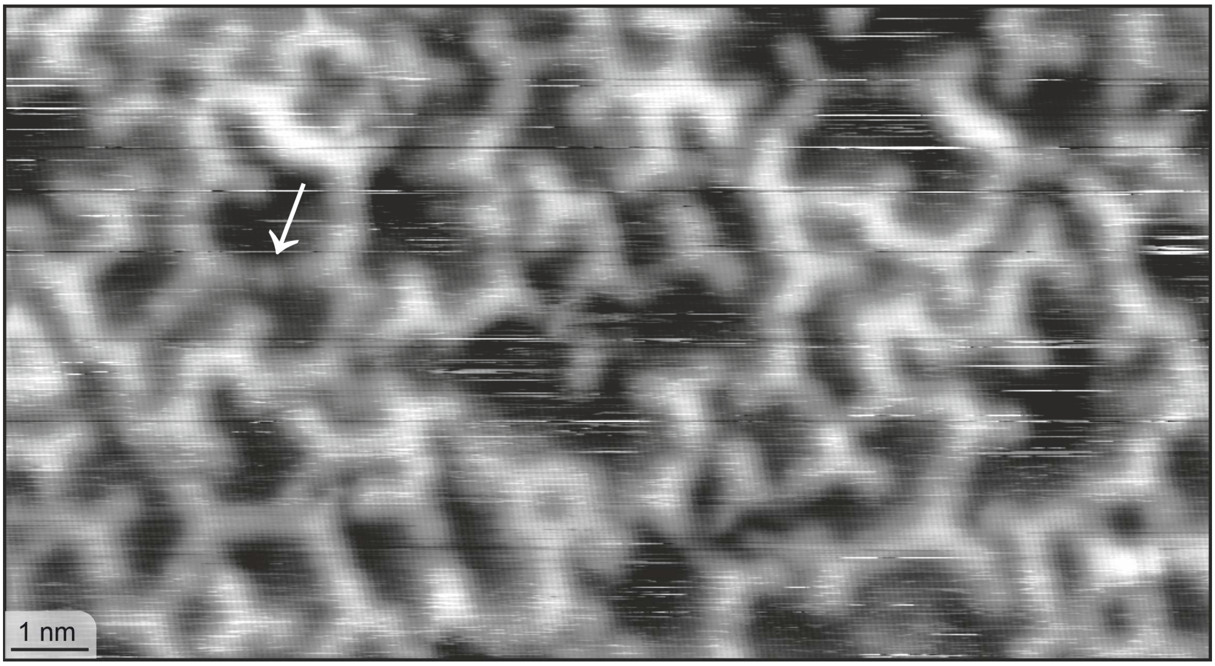


**Figure S4:** Polymeric network comprising decarboxylated BTB molecules. The circular object (highlighted by arrow) is an Ag adatom bound to phenyl radicals. Scanning parameters: 0.8 V, 100 pA.

## 3. Compact δ-BTB layer and pentacene-BTB mixed phases on Ag(100)

The robustness of the compact δ-BTB layer was also tested on Ag(100) to show the large insensitivity of the BTB layer to a surface termination. Figure S5 shows submonolayer coverage of δ-BTB on Ag(100) with a close-packed structure similar to Ag(111). The deprotonation was obtained by annealing the sample with as-deposited BTB to 180–200 °C. To obtain the full δ-BTB layer, the initial coverage was increased accordingly. The large-scale STM image of the compact δ-BTB layer on Ag(100) is given in Figure S6.

Next, by the deposition of 0.5 ML of pentacene and 0.5 ML of BTB molecules on Ag(100) and subsequent annealing, the pentacene–BTB mixed phase with a corresponding 1:1 ratio was formed. The STM and LEEM results are summarized in Figure S7. The measured diffraction and its model made in ProLEED Studio are shown in Figure S7b and c, respectively. Figure S7d provides an STM image of the mixed phase with a marked unit cell. The modeled structure and the superlattice arrangement in the matrix notation with respect to the substrate lattice are depicted in Figure S7e and f, respectively.

Similar to Ag(111), the formation of distinct mixed phases on Ag(100) is observed for different initial ratios of deposited molecules. In a separate experiment, the annealing of a mixture with different initial pentacene:BTB ratio led to the formation of two distinct molecular phases at the same time. The first is the 1:1 phase described above, and the second is 3:4. The STM and LEEM analysis is shown in Figure S8. Both molecular phases are clearly visible in the bright-field image (Figure S8a). The diffraction in Figure S8b is therefore composed of both phases, which are distinguished in the model (Figure S8c) by red (3:4 phase) and black (1:1 phase) spots. The STM image of the 3:4 phase with marked unit cell and its model are shown in Figure S8d and Figure S8e, respectively. Figure S8f shows the superlattice model of the 3:4 phase in the matrix notation.

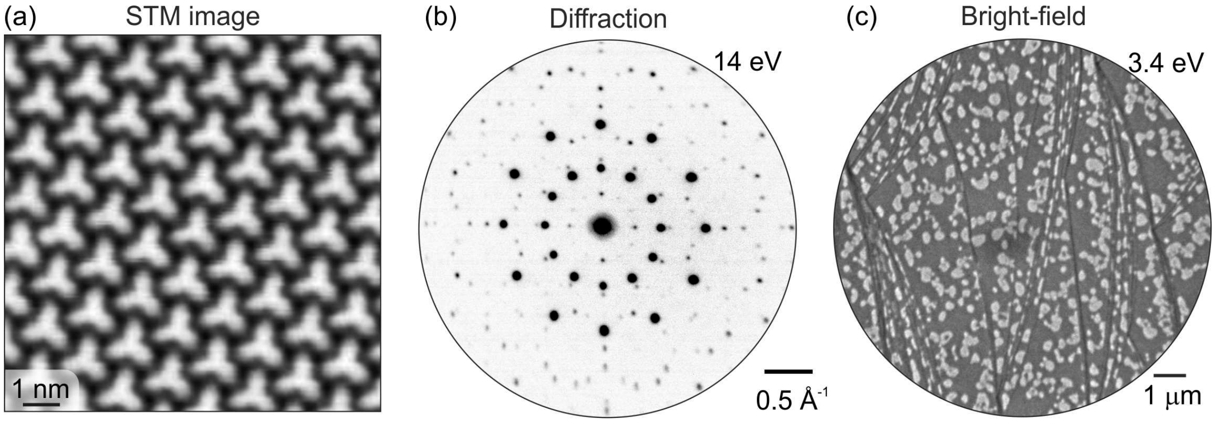


**Figure S5:** Submonolayer coverage of δ-BTB on Ag(100). (a) STM, (b) diffraction, and (c) LEEM bright-field image. Scanning parameters in (a): 1.0 V, 50 pA.

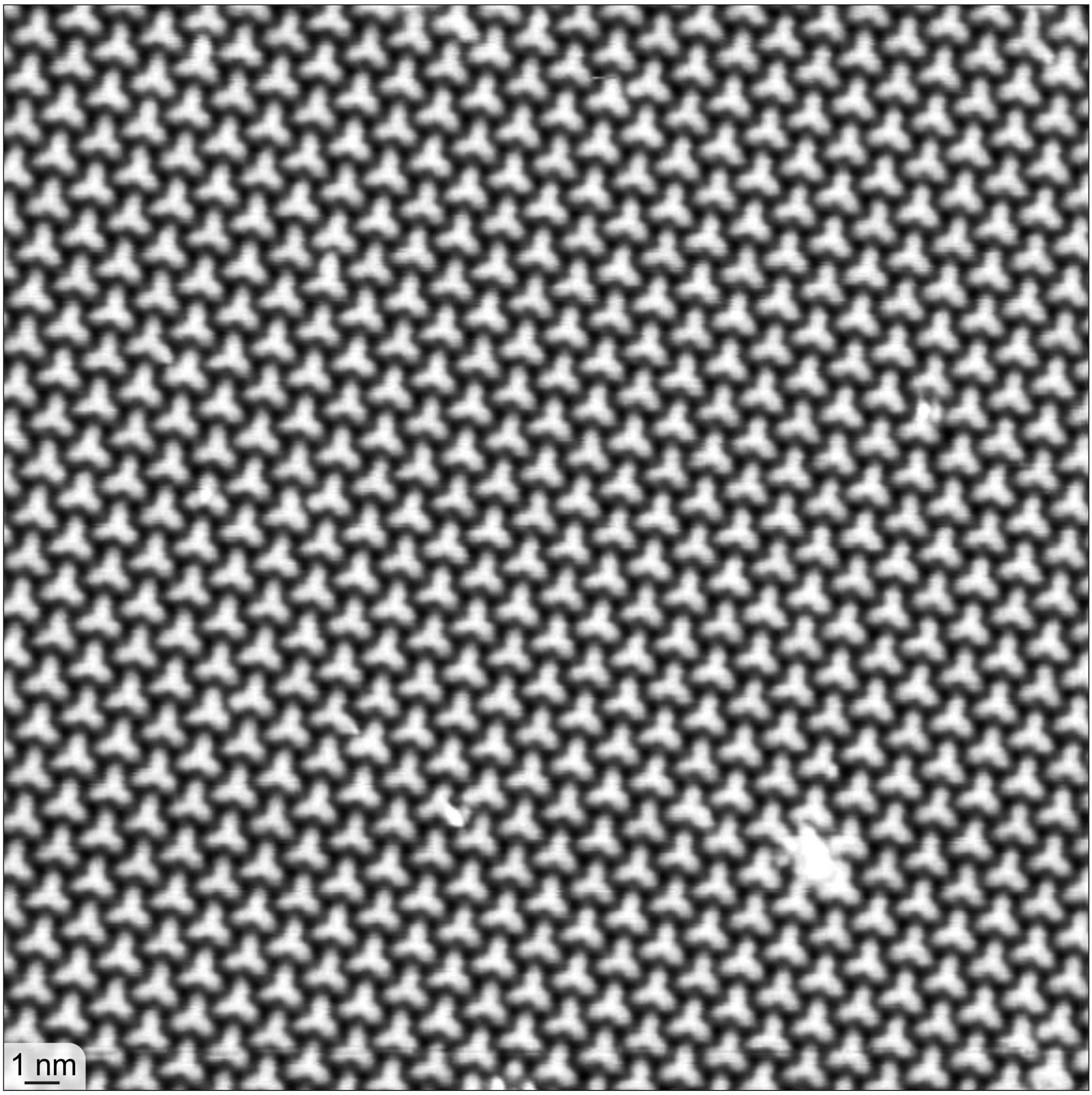


**Figure S6:** Compact δ-BTB layer on Ag(100) surface. Scanning parameters: 1.0 V, 50 pA.

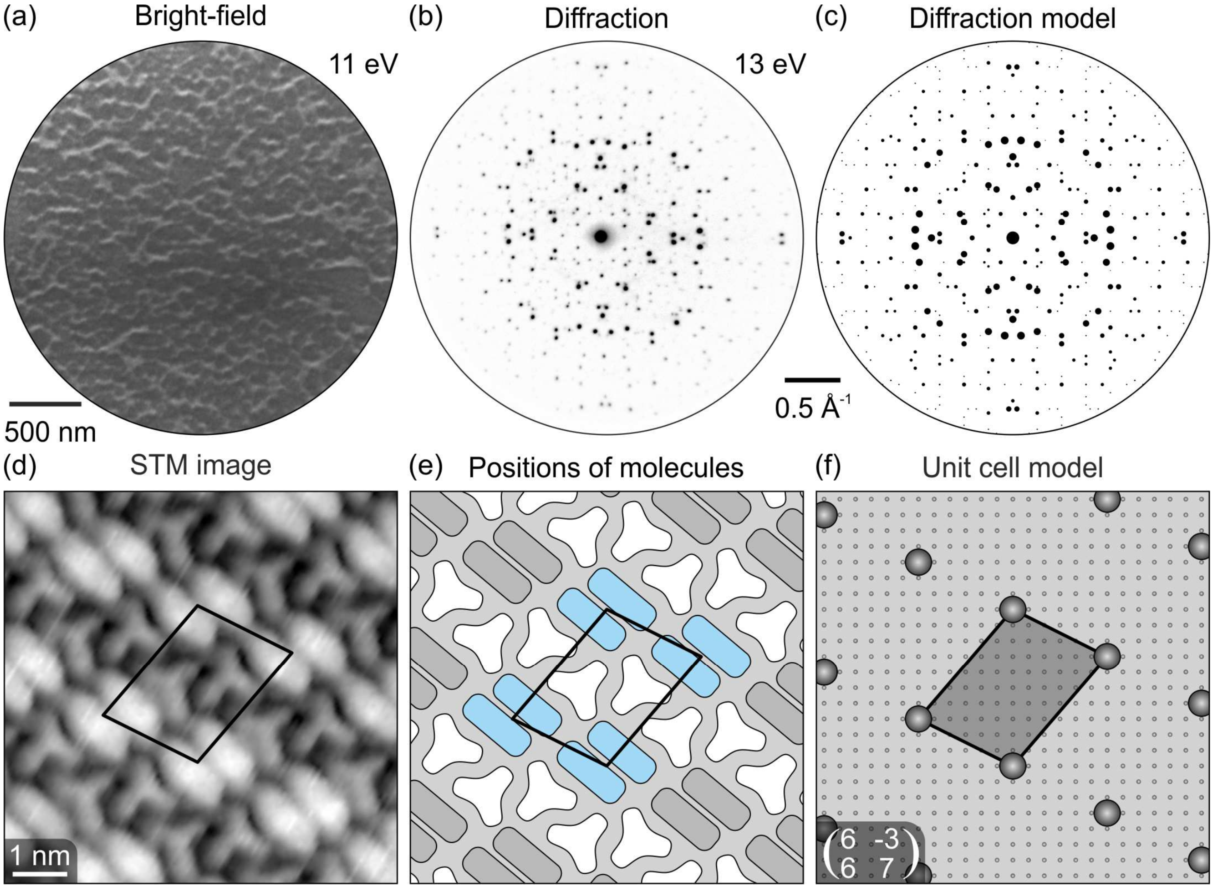


**Figure S7:** STM and LEEM analysis of pentacene-BTB mixed phase with 1:1 ratio of pentacene and BTB at submonolayer coverage. (a) Bright-field LEEM, (b) diffraction, and (c) the modeled diffraction. (d) STM image of the 1:1 structure, (e) modeled positions of the molecules, and (f) their corresponding superlattice description in the matrix notation.

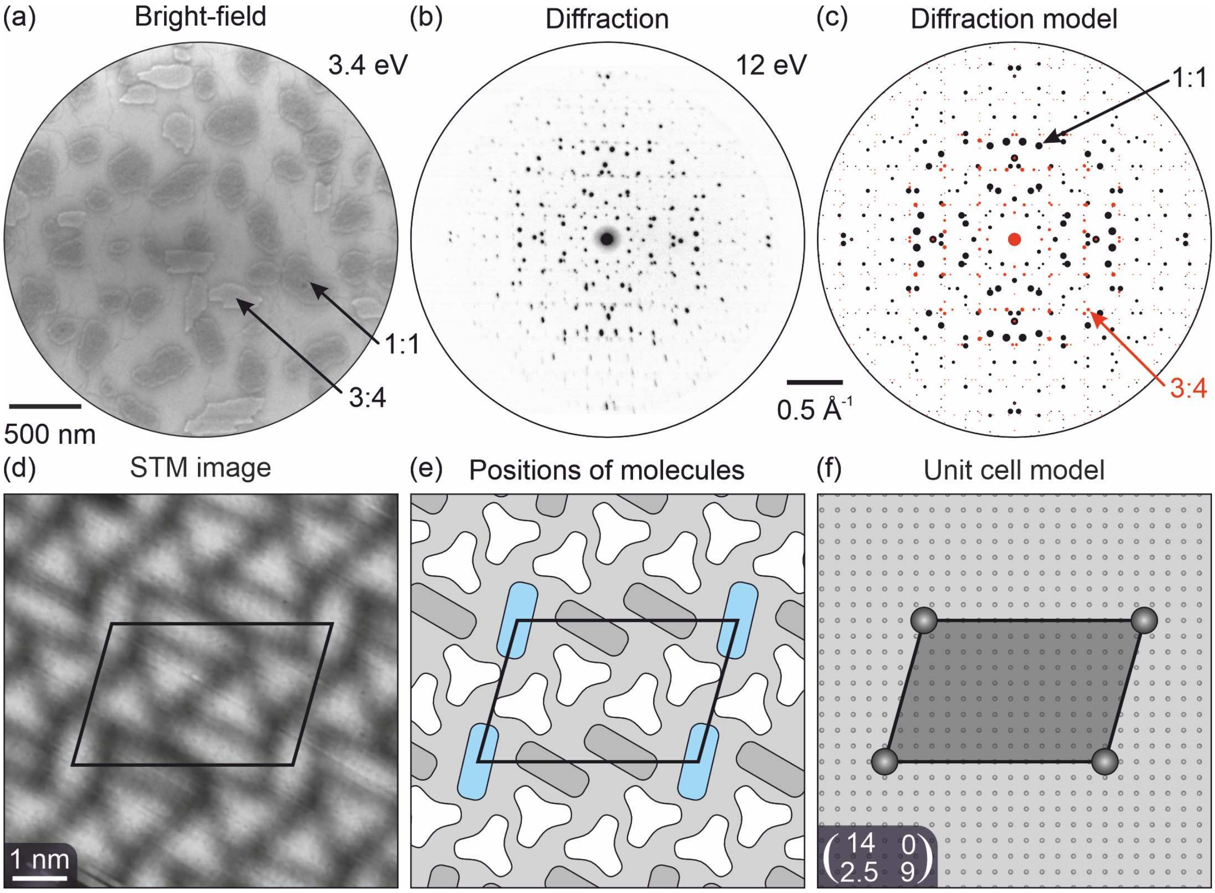


**Figure S8:** STM and LEEM analysis of pentacene-BTB mixed phase with 3:4 pentacene to BTB ratio at submonolayer coverage. (a) Bright-field LEEM, (b) diffraction, and (c) the modeled diffraction shows the combination of 3:4 and 1:1 mixed phases both present on the surface. The 3:4 phase diffraction model is red in (c). (d) STM image of the 3:4 structure, (e) modeled positions of the molecules, and (f) their corresponding superlattice description in the matrix notation.

## 4. Laboratory XPS analysis of the compact δ-BTB layer

The O 1s and C 1s photoemission spectra of the fully deprotonated compact δ-BTB layer on Ag(111) substrate measured in situ by a laboratory XPS (Figure S9) are consistent with the synchrotron measurements. The photoelectron spectrum for the O 1s region shows a single peak at binding energy (BE) of 530.5 eV, consistent with the fully deprotonated BTB on the surface. The C 1s region comprises a peak component associated with phenyl rings at BE 284.2 eV and a carboxyl peak at BE ~287.5 eV, again fully consistent with synchrotron radiation spectra.

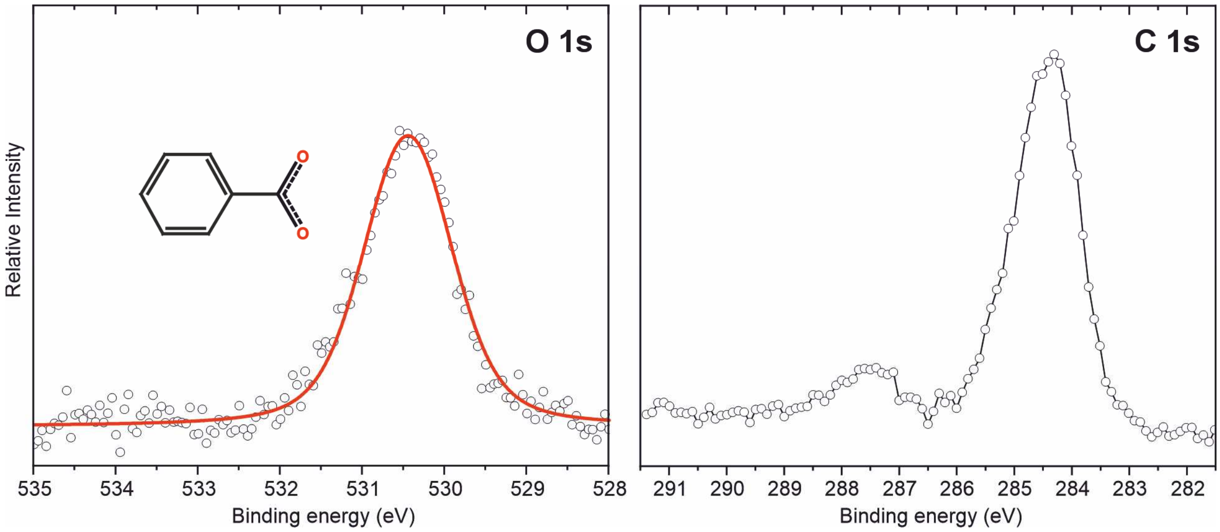


**Figure S9:** O 1s (left) and C 1s (right) measured by laboratory XPS on compact δ-BTB layer on Ag(111).

## 5. Supplementary STM figures of δ-BTB on Ag(111)

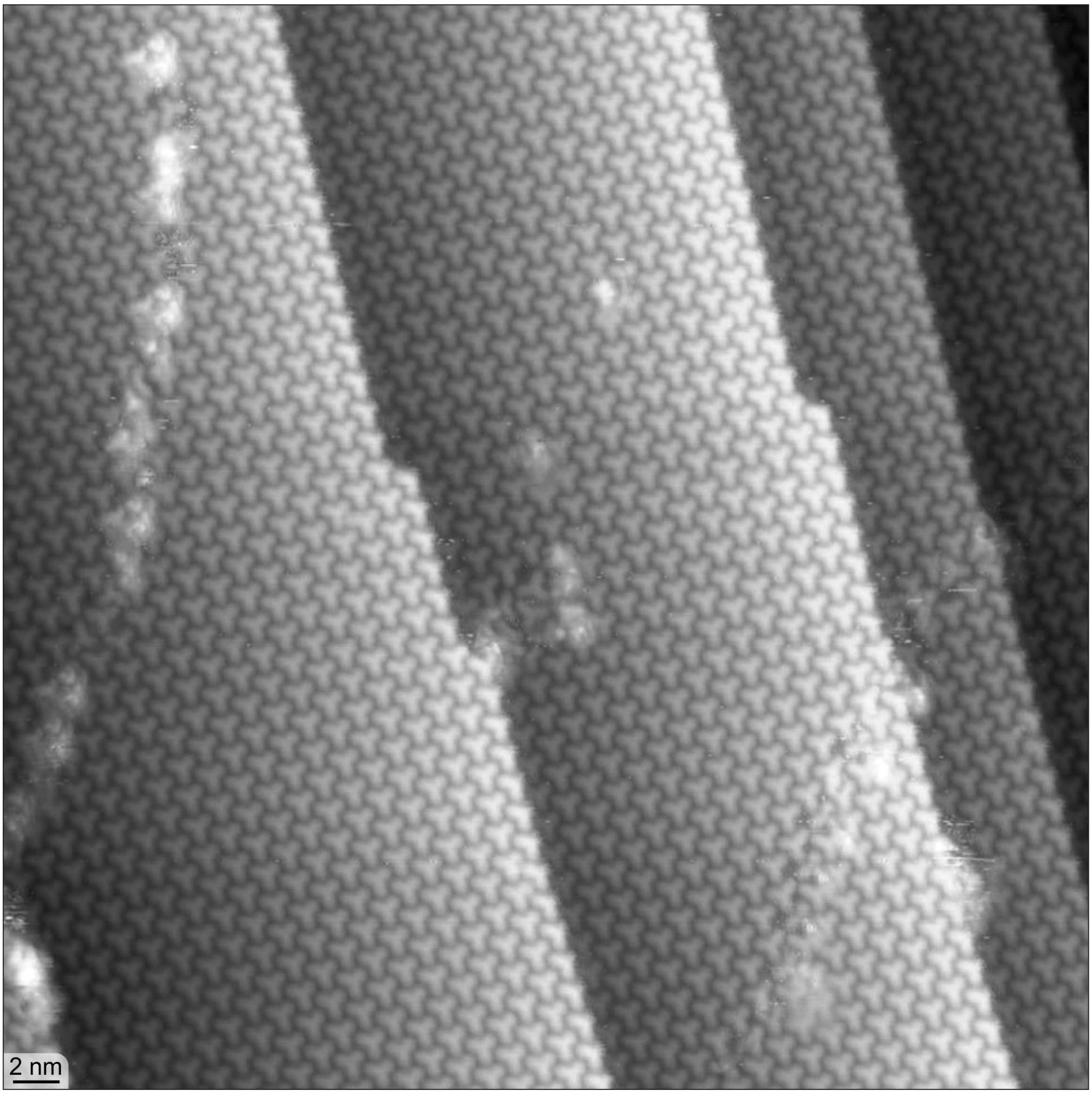


**Figure S10:** Large-scale STM image of compact δ-BTB layer on Ag(111) seamlessly extending over several terraces. Scanning parameters: 1.4 V, 50 pA.

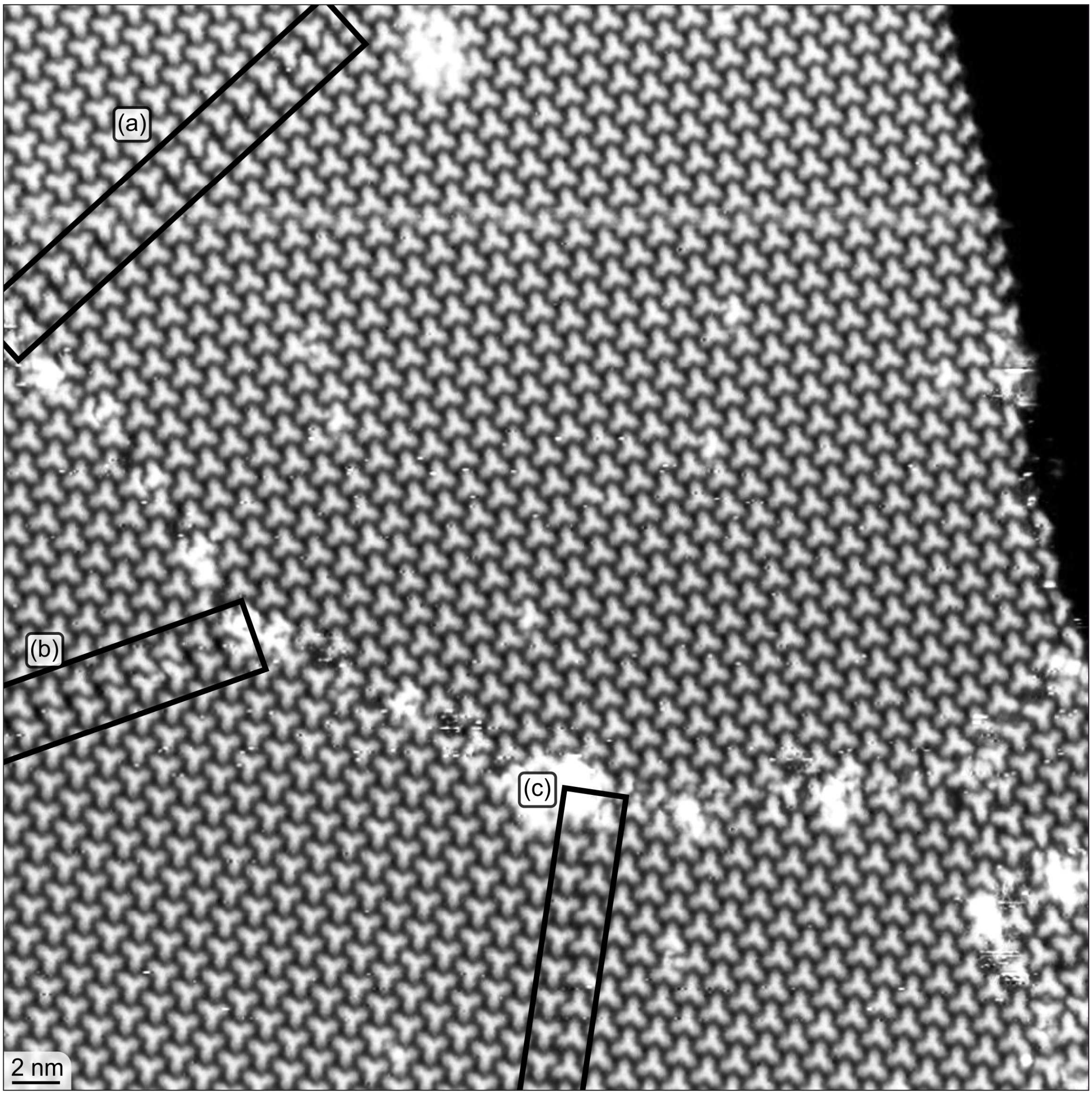


**Figure S11:** STM image of compact δ-BTB layer on Ag(111). Domain boundaries are marked with black rectangles (a–c). Scanning parameters: 1.4 V, 50 pA.

## 6. Comparison of δ-BTB unit cell on Ag(100) and Ag(111)

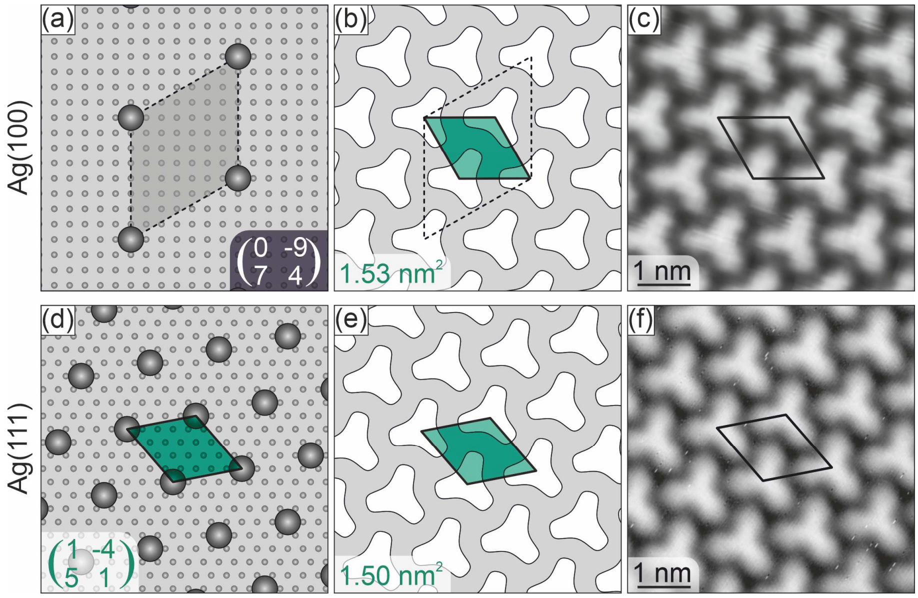


**Figure S12:** Comparison of the structure of the compact δ-BTB layer for (a–c) Ag(100) and (d–f) Ag(111). (a) The alignment of the commensurate δ-BTB unit cell with the Ag(100) lattice. (b) Position of the δ-BTB molecules within the unit cell. (c) The STM image of the δ-BTB layer on Ag(100) with highlighted apparent unit cell given in green in (b). (d) The commensurate unit cell (green) for δ-BTB on Ag(111). (e) Position of the δ-BTB molecules within the unit cell. (f) STM image confirming the presented model.

## 7. Stable overlayers of HM-TP and HAT-CN

In addition to pentacene, we have also tested two other organic semiconductors, i.e., 2,3,6,7,10,11-hexamethoxytriphenylene (HM-TP) and 1,4,5,8,9,12-hexaazatriphenylene hexacarbonitrile (HAT-CN), to show the generality of the robustness of the compact δ-BTB layer with respect to mixing with deposited overlayers.

Synchrotron radiation photoelectron spectroscopy measurements on a compact δ-BTB layer on Ag(111) acquired before deposition, after deposition of submonolayer coverage and subsequent annealing at 220 °C are given in Figure S13 for HAT-CN and Figure S14 for HM-TP. In the case of HAT-CN, we observe a new peak at 287.2 eV after HAT-CN deposition; after annealing, its intensity decreases, but HAT-CN is not removed completely below the decarboxylation threshold of BTB. As HAT-CN does not contain oxygen atoms, we observe a decrease in the O 1s signal after HAT-CN deposition due to covering the δ-BTB layer and attenuation of the signal from the lower-lying layers. After annealing to 220 °C, the O 1s peak intensity slightly increases. Importantly, we do not observe any significant changes in both peak position and shape that would indicate changes in the δ-BTB layer, i.e., its interaction with HAT-CN.

In the case of HM-TP, our photoelectron spectroscopy measurements show a similar result. The spectra in Figure S14 show that after HM-TP deposition, there is an additional signal in the C 1s spectrum and an additional single peak at 533.9 eV in the O 1s spectrum. The peak associated with carboxylate oxygen is only slightly reduced in intensity, and we do not observe any changes in its position or shape. After annealing at 220 °C, both C 1s and O 1s spectra almost change back to their original appearance; however, a small portion of HM-TP molecules remain on the surface. Also, in this case, we do not observe any signs of change in peaks associated with the δ-BTB layer.

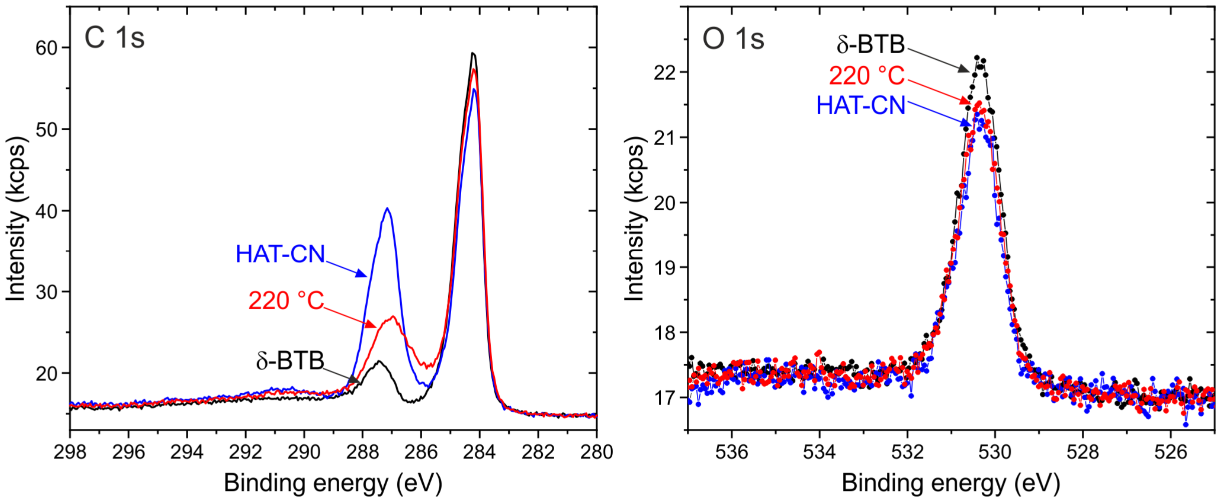


**Figure S13:** C 1s and O 1s spectra obtained by synchrotron radiation photoelectron spectroscopy for HAT-CN deposited on compact δ-BTB layer on Ag(111). The spectra measured on a compact δ-BTB layer are marked as "δ-BTB", spectra measured after a deposition "HAT-CN" and after subsequent annealing to 220 °C "220 °C". The spectra are vertically shifted to match the background at the low binding energy side of each peak.

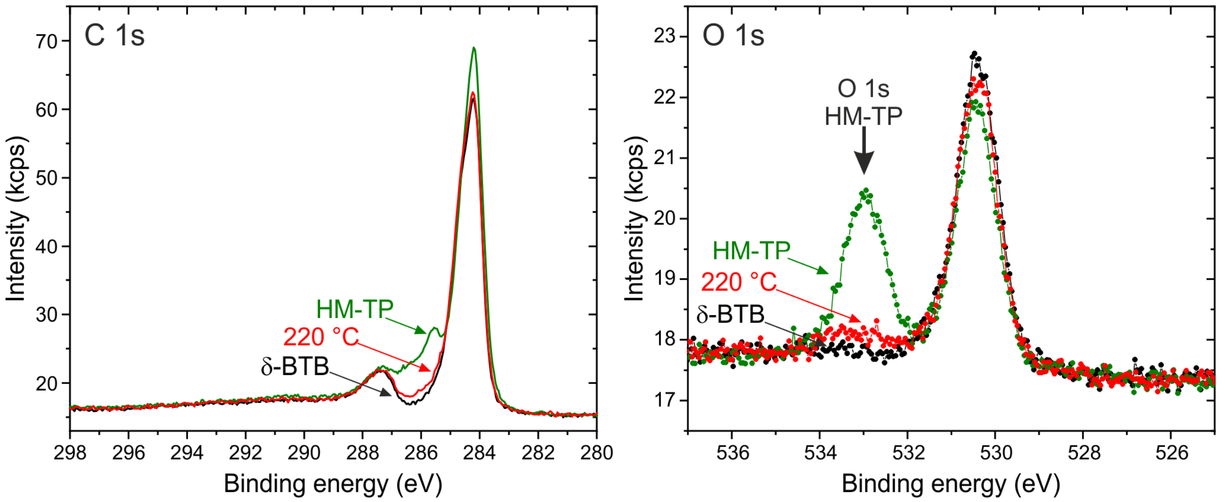


**Figure S14:** C 1s and O 1s spectra obtained by synchrotron radiation photoelectron spectroscopy for HM-TP deposited on compact δ-BTB layer on Ag(111). The spectra measured on a compact δ-BTB layer are marked as "δ-BTB", spectra measured after a deposition "HM-TP" and after subsequent annealing to 220 °C "220 °C". The spectra are vertically shifted to match the background at the low binding energy side of each peak. A vertical arrow marks the O 1s peak associated with HM-TP.

LEEM analysis of the δ-BTB layer on the Ag(111) surface before and after HAT-CN deposition is summarized in Figure S15. After the submonolayer deposition, large dark areas with different microdiffraction patterns appeared in the bright-field images (Figure S15b). Therefore, we associate these areas with the ordered HAT-CN overlayer. In line with the XPS results, annealing of the substrate up to 220 °C led to a decreased area covered by the HAT-CN islands. During the experiment, we did not observe any signs of disruption of the compact δ-BTB layer or formation of mixed HAT-CN–BTB phases. However, as HAT-CN remains on the surface, an unambiguous proof of not mixing cannot be provided solely by LEEM measurements.

Similarly, we have tested HM-TP, but in this case, the LEEM analysis is complicated by the fact that HM-PT has the same unit cell as the δ-BTB layer. Also, in this case, we did not observe any signs of disruption of the compact δ-BTB layer.

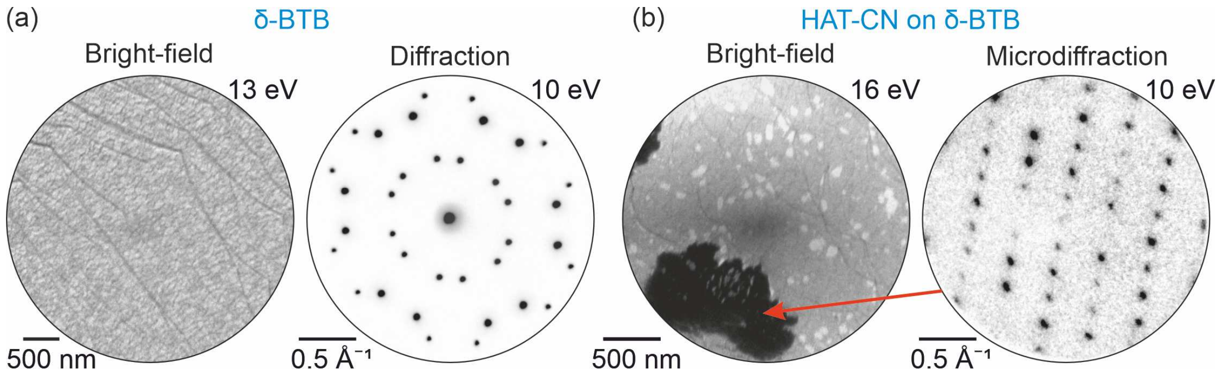


**Figure S15:** LEEM bright-field and diffraction pattern images before (a) and after (b) HAT-CN deposition. Microdiffraction images taken from the HAT-CN overlayer indicate different periodicity of HAT-CN islands.

## 8. The wheel-like pentacene-BTB mixed phase on Ag(111)

In addition to the 1:1 pentacene-BTB mixed phase discussed in the main text, 2:1 phase was also obtained for a different initial pentacene:BTB ratio. The bright-field LEEM given in Figure S16a shows bright islands of the mixed phase on Ag(111) substrate. Figures S16b and c show the mixed phase diffraction and its model. The size and orientation of the unit cell fit the real space structure observed in the STM image of the mixed phase given in Figure S16d. The unit cell consists of 6 pentacene and 6 δ-BTB molecules arranged into a wheel, and 6 additional pentacenes at its periphery (see Figure S16e). The position of the unit cell with respect to the substrate lattice is given in Figure S16f.

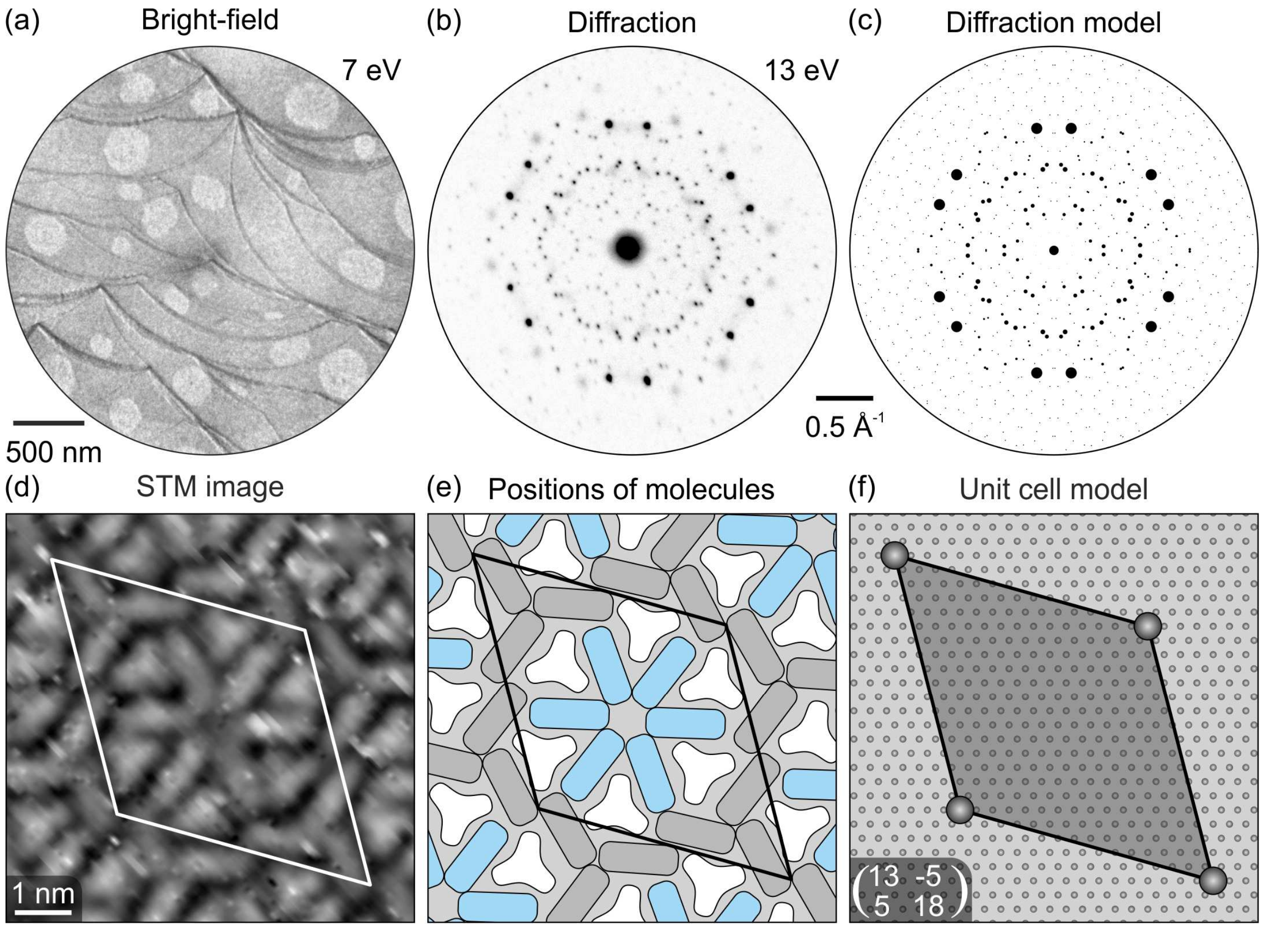


**Figure S16:** The wheel-like pentacene-BTB mixed phase on Ag(111) with 2:1 ratio of pentacene:BTB. (a) bright field LEEM, (b) diffraction pattern, and (c) modeled diffraction of the mixed phase. (d) STM image with unit cell marked by a black rhombus. Scanning parameters: 1.1 V, 50 pA. (e) Schematics of positions of the molecules within a unit cell. (f) Superstructure unit cell with respect to the substrate.

## 9. Gas-phase reference for calculated adsorption energies

The stability of the δ-BTB layer is evaluated in the main text with respect to direct desorption of an ionized BTB molecule. An alternative desorption process of a BTB molecule from the δ-BTB layer consists of a full protonation of a BTB molecule, followed by its detachment from the molecular layer into a vacuum. Here, a fully protonated gas-phase BTB molecule would serve as the reference state. In the following we demonstrate that the δ-BTB phase is favored over the intermixed phase even in this case, although the quantitative outcomes are strongly functional dependent. In this scenario, the adsorption energy per unit area $\gamma$ is a function of a hydrogen chemical potential:

$$\gamma\left(\mu_{\mathrm{H_2}}\right) = \frac{E_{\mathrm{int}} - E_{\mathrm{sub}} + \frac{3}{2}\left(E_{\mathrm{H_2}} + \mu_{\mathrm{H_2}}\right) - E^{\mathrm{gas}}_{\mathrm{H_3BTB}}}{S}, \qquad \text{(S1)}$$

where $E_{\mathrm{int}}$ and $E_{\mathrm{sub}}$ are total energies of an interface and a Ag(111) substrate system, $E_{\mathrm{H_2}}$ and $E^{\mathrm{gas}}_{\mathrm{H_3BTB}}$ are gas phase energies for a hydrogen molecule and for a (fully protonated) BTB molecule, $\mu_{\mathrm{H_2}}$ is a hydrogen molecule chemical potential, and $S$ stands for an area of the supercell.

Calculated adsorption energies per unit area employing PBE-D3 and optB86b functionals are depicted in Figure S17. As illustrated, under experimental conditions related to hydrogen chemical potential of –1.07 eV, the δ-BTB phase is calculated to be in both cases more stable than the intermixed phase. Nonetheless, the calculated critical chemical potentials and transition pressures exhibit significant dependence on the DFT functional employed. The transition occurs at –0.89 eV (PBE-D3) and at –0.49 eV (optB86b), which corresponds to the hydrogen transition pressure of ~$10^{-7}$ mbar and ~1 mbar, respectively. We also note that the phase diagram is not complete, as it does not contain semi-protonated and protonated BTB phases. However, given the seven orders of magnitude difference in the calculated critical pressure yielded by these functionals, we conclude that DFT does not provide a deeper understanding of the phase stability as a function of the hydrogen chemical potential.

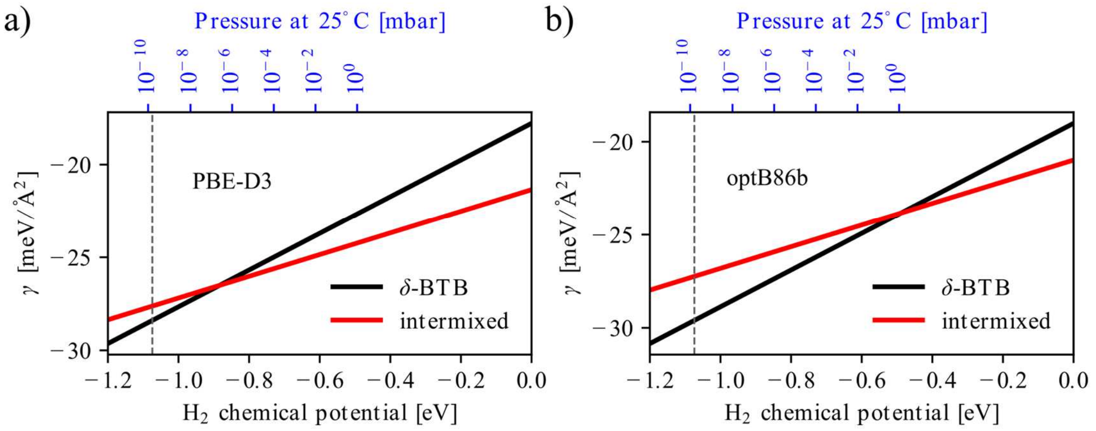


**Figure S17:** Calculated surface energies of the δ-BTB and intermixed phases as a function of a hydrogen chemical potential and a hydrogen pressure at 25 ˚C using (a) PBE-D3 functional and (b) optB86b functional. A gray dashed line marks a chemical potential under experimental conditions.

## 10. Supplementary DFT structures of molecular monolayers on Ag(111) substrate

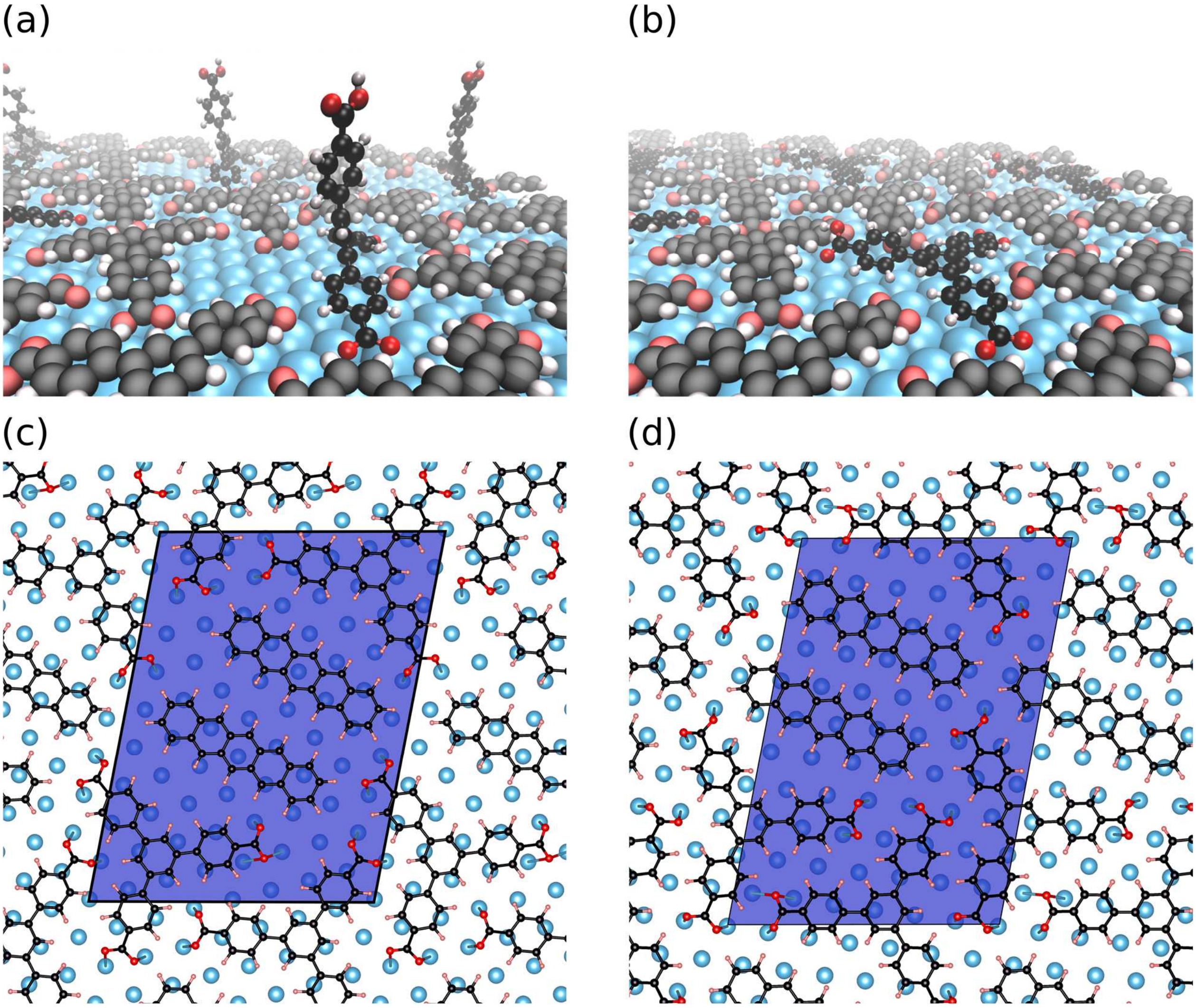


**Figure S18:** Models used for DFT calculations. (a) and (b) partially protonated BTB molecule in the standing-up (a) and in the lying (b) configurations. The BTB molecule being detached is rendered with higher contrast. (c) Structural model for the intermixed phase with the unit cell obtained from diffraction. (d) The same structure with the unit cell giving the highest stability.
